# HosA-mediated epigenetic regulation of growth, virulence, and secondary metabolism in *Aspergillus fumigatus*

**DOI:** 10.1080/21505594.2026.2655064

**Published:** 2026-04-02

**Authors:** Zhengyu Zhou, Yuqi Zhu, Renwei Gao, Yamei Wang, Yin Chen, Ling Lu, Yuanwei Zhang

**Affiliations:** a Jiangsu Key Laboratory for Pathogens and Ecosystems, Jiangsu Engineering and Technology Research Centre for Microbiology, College of Life Sciences, Nanjing Normal University, Nanjing, China; b Department of Pharmacology and Toxicology, R. Ken Coit College of Pharmacy, University of Arizona, Tucson, AZ, USA

**Keywords:** *Aspergillus fumigatus*, histone deacetylation, secondary metabolism, environmental adaptation, virulence

## Abstract

*Aspergillus fumigatus* is a major opportunistic fungal pathogen whose pathogenic success relies on tight coordination between growth, metabolism, and environmental adaptation. Secondary metabolites (SMs) contribute to virulence and ecological fitness but impose substantial metabolic costs. Here, we identify the histone deacetylase HosA as a chromatin-level regulator that promotes fungal growth and virulence while repressing secondary metabolism. Genome-wide ChIP-seq revealed no significant global changes in acetylation upon *hosA* deletion but revealed locus-specific increases across the fumagillin/pseurotin supercluster and the Velvet complex genes. Transcriptomic analyses at 28°C and 37°C demonstrated temperature-dependent regulation of SM clusters that correlates with reduced *hosA* expression at lower temperatures. Loss of HosA further enhanced fumagillin-dependent inhibition of Gram-positive bacteria, highlighting a trade-off between growth and chemical defense. Together, our findings uncover a chromatin-based regulatory mechanism that coordinates fungal growth, virulence, and secondary metabolism, providing insights into *A. fumigatus* pathogenic fitness and potential antifungal target development.

## Introduction

*Aspergillus fumigatus* is a ubiquitous saprophytic mold commonly found in soil, decaying organic matter, and indoor environments. Despite its environmental origin, it is the primary causative agent of invasive aspergillosis (IA), a life-threatening infection that primarily affects immunocompromised individuals [1,2]. Due to the increasing prevalence of antifungal resistance and limited therapeutic options, *A. fumigatus* has been classified as a “critical priority” fungal pathogen by the World Health Organization [3]. Although capable of causing severe disease, A. fumigatus is considered an opportunistic pathogen [4,5]. It causes disease primarily in immunocompromised hosts, and its pathogenicity is thought to be an incidental consequence of traits evolved for environmental survival such as resistance to thermal and oxidative stress, nutrient limitation, and microbial competition [6–8]. These features collectively enable *A. fumigatus* to be a successful opportunistic pathogen in the human host. It is therefore essential to elucidate the molecular mechanisms that underlie both its pathogenic potential and its ability to survive across diverse environments.

The ability to produce a broad spectrum of secondary metabolites (SMs) plays a central role in A. fumigatus ecological fitness and host–pathogen interactions [9,10]. These metabolites, including gliotoxin and fumagillin, modulate host immune responses, inhibit microbial competitors [11,12], and enhance survival under stress [13]. However, SM production is energetically expensive, requiring the fungus to balance biosynthesis with growth and development depending on environmental conditions. This trade-off is particularly evident in temperature-dependent SM regulation, where many fungal species silence biosynthetic gene clusters (BGCs) above 37°C, a phenomenon observed for decades in industrial and laboratory settings. The transcription of BGCs is tightly regulated in response to environmental and developmental signals, including nutrient status, light, temperature [14,15], and microbial challenge by transcription factors or global regulators like the Velvet complex (LaeA, VeA, VelB) [16–18] and chromatin modifications [19,20]. Yet, the molecular mechanisms by which environmental signals such as temperature or microbial interactions dynamically modulate these regulators remain poorly understood.

Histone deacetylases (HDACs) promote chromatin condensation and limit transcriptional accessibility, thereby repressing gene expression within biosynthetic gene clusters (BGCs) [21–23]. In fungi, HDACs are evolutionarily conserved and classified into three major classes: class I and II (Zn^2+^-dependent) and class III (NAD^+^-dependent sirtuins) [24,25]. These enzymes play important roles in regulating fungal development, metabolism, and host interaction. For example, RpdA is essential for viability in several filamentous fungi including *A. fumigatus*, *Aspergillus nidulans,* and *Magnaporthe oryzae* [26–28]. Its downregulation or conditional repression causes severe developmental defects, and complete disruption is lethal. In pathogens such as *Fusarium graminearum*, *M. oryzae,* and *Beauveria bassiana*, Hos2 influences gene expression of effector, oxidative stress tolerance, morphogenesis, and virulence [29–31]. In filamentous fungi, secondary metabolism is closely tied to developmental stages and is often subject to temperature-dependent control [32,33]. HDACs, in particular, have emerged as key repressors of SM gene expression across diverse fungal species. However, despite their central regulatory functions, HDACs are generally considered constitutively expressed chromatin modifiers. Whether HDAC expression itself responds to environmental signals such as temperature shifts or microbial encounters remains largely unexplored.

To address this gap, we systematically identified and characterized HDACs in *A. fumigatus* and uncovered the class I HDAC-HosA as a bifunctional regulator that promotes growth and virulence while repressing secondary metabolism. We found that *hosA* expression is higher at 37°C than at 28°C, a pattern that corresponds to the absence of fumagillin and pseurotin at 37°C and their accumulation at 28°C. Our findings position HosA as a key regulator that coordinates developmental and metabolic outputs, providing new insights into fungal adaptability and potential antifungal strategies.

## Results

### Systematic identification of HDACs reveals HosA as a key regulator of fungal growth and secondary metabolism

**Figure 1.**
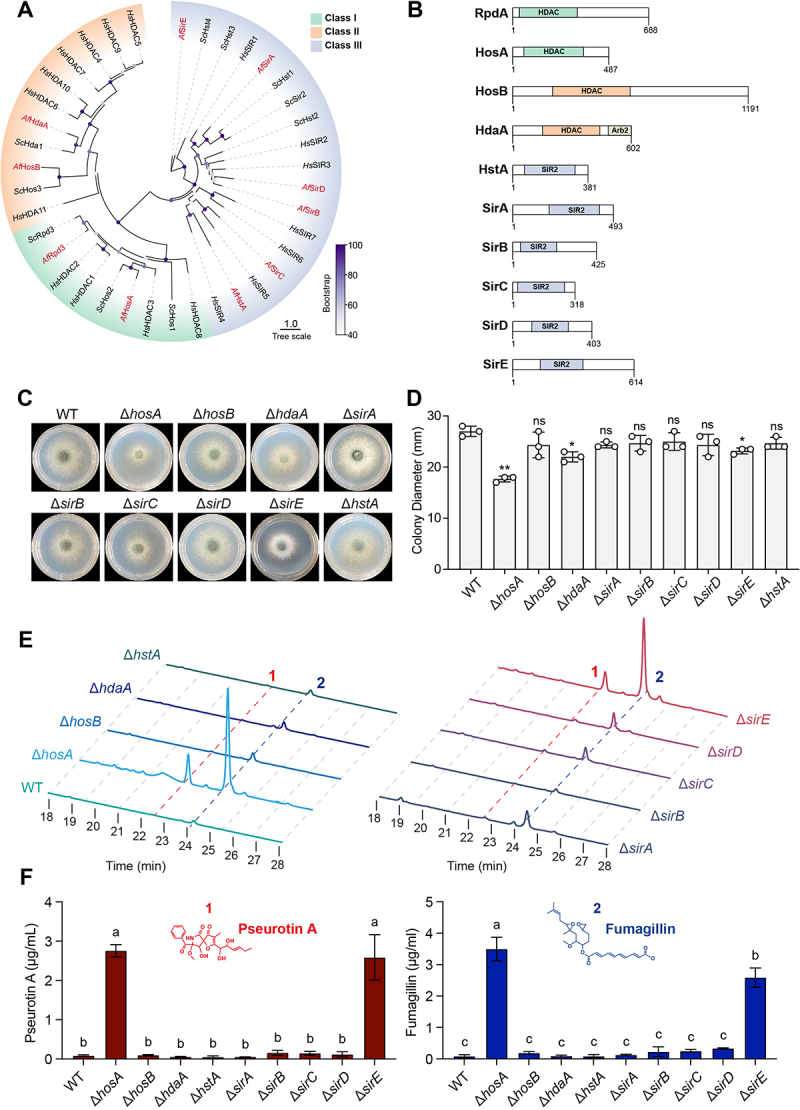
Systematic characterization of histone deacetylases in *A. fumigatus* identifies HosA as a central regulator of fungal growth and secondary metabolism. (A) Phylogenetic tree of histone deacetylase (HDAC) orthologs. A neighbor-joining phylogenetic tree was constructed using protein sequences of HDAC from *A. fumigatus* (*Af*), *S. cerevisiae* (*Sc*) and *H. sapiens* (*Hs*). Predicted *A. fumigatus* HDACs are highlighted in red. (B) Domain architecture of predicted HDACs in *A. fumigatus* based on SMART database analysis. (C) Colony morphology of HDAC deletion mutants grown on minimal medium (MM) for 48 h at 37°C. (d) Quantification of colony diameters for HDAC deletion mutants after 48 h of incubation on MM. Data represent the mean ± standard deviation (sd) from three independent experiments. Statistical significance was determined by one-way ANOVA compared to the wild-type strain. **p < 0.01; *p < 0.05; ns, not significant. (E) HPLC chromatograms of culture supernatants from wild-type strain and HDAC deletion mutants, following 72 h fermentation at 37°C in minimal medium. Peaks 1 and 2 correspond to pseurotin a and fumagillin, respectively. (F) Quantification of pseurotin a (left) and fumagillin (right) in the indicated strains. Data represent mean ± sd from three independent experiments. Statistically significant differences were determined by one-way ANOVA (*p* < 0.01). Different letters indicate significant differences.

To systematically elucidate the roles of potential HDACs in regulating fungal development and secondary metabolism in *A. fumigatus*, we first identified candidate HDACs by performing BLASTp alignment of known HDAC sequences from *Saccharomyces cerevisiae* and *Homo sapiens* against the *A. fumigatus* A1163 database. Phylogenetic and domain analyses revealed 10 potential HDACs in *A. fumigatus* distributed across Class I, Class II, and Class III (Figure 1(A,B)). To investigate the functional roles of the predicted HDACs in *A. fumigatus*, individual knockouts of the identified HDAC genes were generated via homologous recombination (Figure S1), we successfully obtained knockout strains for all candidates except *rpdA*, consistent with previous reports indicating its essentiality for fungal viability [34,35]. Δ*hosA*, Δ*hdaA* and Δ*sirE* mutants exhibited marked growth defects and reduced conidiation compared to the wild-type strain (Figure 1(C)). Notably, the Δ*hosA* mutant exhibited the most pronounced growth defect (Figure 1(D)). Given the pronounced developmental phenotypes observed in several HDAC mutants, we next examined whether these HDACs also influence the production of secondary metabolites. We focused on the production of two representative metabolites, fumagillin and pseurotin A, which are derived from a tightly intertwined biosynthetic supercluster [13,36,37] and associated with host-pathogen interactions [38–40]. Following 72 h culture in liquid minimal medium at 37°C, HPLC analysis of culture supernatants revealed that the Δ*hosA* and Δ*sirE* mutants displayed two prominent peaks that are absent in the wild-type strain (Figure 1(E)). LC-MS confirmed these compounds as fumagillin and pseurotin A, which are two well-characterized metabolites synthesized from the intertwined fumagillin/pseurotin supercluster (Figure S2). Quantitative analysis showed that the Δ*hosA* and Δ*sirE* mutants produced significantly higher levels of both metabolites compared to the wild-type strain (Figure 1(F)). Among the HDAC mutants, Δ*hosA* exhibited the most severe phenotypes in terms of both impaired growth and increased metabolite production, suggesting a critical role in coordinating fungal development and secondary metabolism.

### *A.* *fumigatus* HosA is required for both fungal growth and virulence

**Figure 2.**
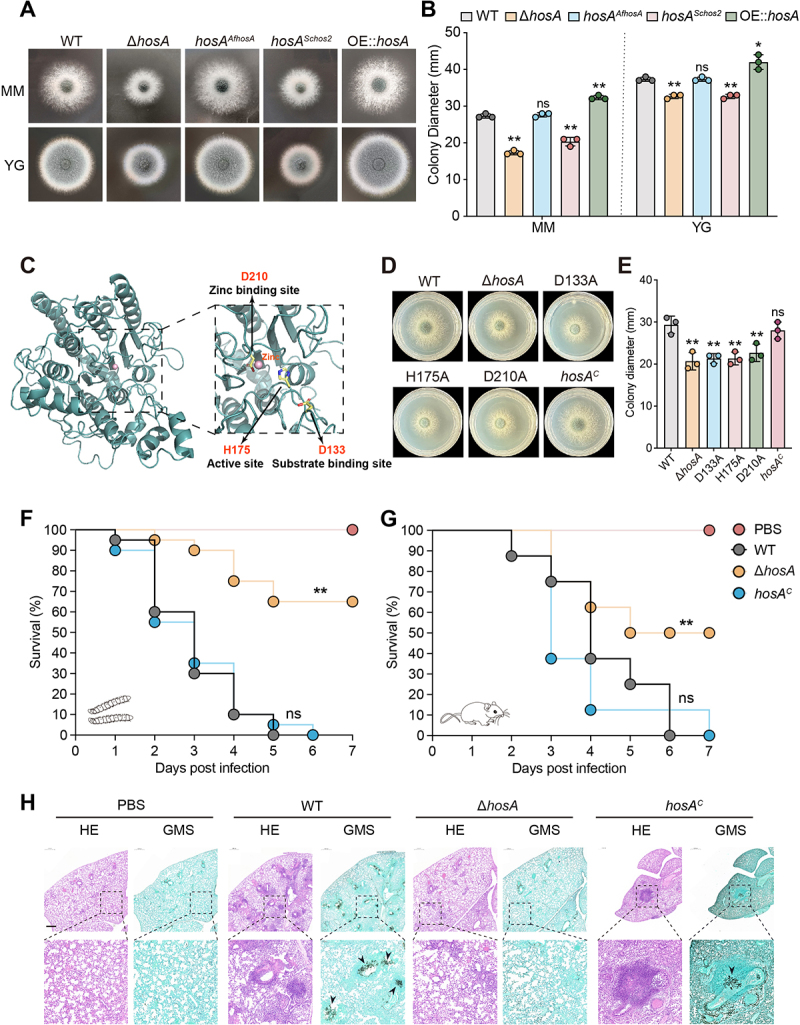
Loss of HosA attenuates *A. fumigatus* virulence in *G. mellonella* and mouse infection models. (A) Colony morphology of wild-type, Δ*hosA* mutant, homologous (*AfhosA*) or heterologous (*Schos2*) complementation and *hosA* overexpression strains grown on minimum medium (MM) and yeast-glucose medium (YG) for 48 h at 37°C. (B) Quantification of colony diameters for the indicated strains on MM and YG media. Data represent the mean ± sd from three independent experiments. Statistical significance was determined by one-way ANOVA. **p < 0.01; *p < 0.05; ns, not significant. (C) Predicted protein structure of *A. fumigatus* HosA highlighting critical residues within its HDAC domain. The amino acid residues D133 (substrate binding), H175 (active site) and D210 (zinc ion binding) amino acid residues within the HDAC domain are highlighted. (D) Colony morphology of *hosA* point-mutation strains on solid minimal medium (MM) for 48 h at 37°C. (E) Quantification of colony diameters for *hosA* point-mutation strains. Data represent the mean ± sd from three independent experiments. Statistical significance was determined by one-way ANOVA compared to the wild-type strain. **p < 0.01. (F) Survival curves of *G. mellonella* larvae infected with *A. fumigatus* wild-type, Δ*hosA* mutant and complementation strains. Sterile phosphate-buffered saline (PBS) was injected as a negative control. Statistical significance of survival differences was assessed using the log-rank (Mantel-Cox) test. **p < 0.01; ns, not significant. (G) Survival curves of neutropenic mice intratracheally infected with *A. fumigatus* wild-type (wt), Δ*hosA* mutant and complementation strains. Statistical significance of survival differences was assessed using the log-rank (Mantel-Cox) test. **p < 0.01; ns, not significant. (H) Histopathological analysis of mouse lung tissues. Lung tissues were harvested from infected mice at 3 days post-infection. Sections were stained with Hematoxylin and Eosin (H&E) to visualize tissue architecture and inflammatory responses and Grocott’s methenamine silver (GMS) staining to detect fungal hyphae (black arrows). Scale bars = 100 µm.

Sequence analysis revealed that HosA harbors a highly conserved histone deacetylase (HDAC) domain, which is broadly shared among fungal orthologs (Figure S3). To determine whether the observed phenotype was caused by *hosA* deletion and to assess its functional conservation, we constructed homologous and heterologous complementation strains by reintroducing *hosA* from *A. fumigatus* (*AfhosA*) and its ortholog from *Saccharomyces cerevisiae* (*Schos2*) into the Δ*hosA* mutant, and successful integration was confirmed by diagnostic PCR (Figure 2(A) and S1). Growth assays on solid minimal medium (MM) and yeast-glucose medium (YG) demonstrated that the growth defects of Δ*hosA* mutant were fully rescued in the homologous complementation strain, but not in the heterologous complementation strain (Figure 2(A,B)), suggesting that the functions of HosA are not entirely conserved across different species. Furthermore, we generated a *hosA* overexpression strain and observed a significant increase in colony diameter compared to the wild-type (Figure 2(A) and S4), further supporting the role of HosA as a positive regulator of fungal growth in *A. fumigatus*.

To further dissect HosA function, we identified three highly conserved residues (D133, H175, D210) within its HDAC domain through sequence alignment and structural modeling (Figure 2(C) and S5A), they were predicted to participate in substrate binding, catalytic activity, and zinc ion coordination, respectively. Site-directed point mutations (D133A, H175A, and D210A) were introduced *in situ* into the endogenous *hosA* locus, with each mutant construct fused to a C-terminal GFP tag. Western blot analysis confirmed that all three mutant fusion proteins are expressed correctly (Figure S5B). Phenotypic characterization revealed that all three mutants exhibited impaired colony growth and increased sensitivity to oxidative (H_2_O_2_) and cell wall stress (Congo red), phenocopying the Δ*hosA* mutant (Figure 2(D,E) and Figure S6). These findings demonstrate that conserved residues within the HDAC domain are critical for the function of HosA.

Given the growth defects in the *hosA* mutant, we assessed its role in virulence using *Galleria mellonella* and murine models. In the *G. mellonella* model, larvae challenged with the Δ*hosA* mutant displayed markedly reduced mortality, remaining below 40% after 7 days, in contrast to 100% mortality by day 5 in the wild-type strain and by day 6 in the complementation strain (Figure 2(F)). Consistent with these findings, the cyclophosphamide-immunosuppressed mouse model also demonstrated significantly reduced virulence in the Δ*hosA* mutant. Mice inoculated with Δ*hosA* showed prolonged survival compared to those infected with either the wild-type or complementation strains (Figure 2(G)). Histopathological analysis further showed that lungs from mice infected with wild-type or complementation strains displayed extensive inflammatory infiltration and hyphal structures, while lungs that infected with Δ*hosA* mutant lacked detectable fungal hyphae (Figure 2(H)). Consistently, fungal burden analysis revealed a 93% reduction in colony-forming units (CFUs) in Δ*hosA*-infected lungs compared to the wild-type and complementation group (Figure S7). Together, these results demonstrate that HosA is indispensable for maintaining full virulence in *A. fumigatus*.

### *A.* *fumigatus* HosA functions as a histone deacetylase

**Figure 3.**
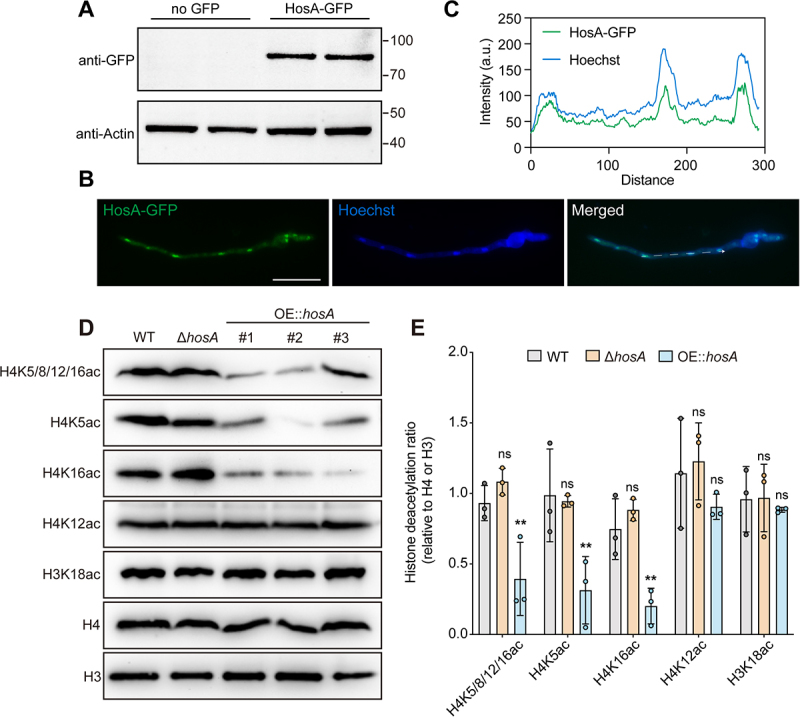
Nuclear localization and histone deacetylase activity of HosA in *A. fumigatus*. (A) Western blot analysis showing the expression of GFP-tagged HosA. The wild-type protein (no GFP tag) served as a negative control. Actin was used as a loading control. (B) Fluorescence microscopy of the GFP-tagged HosA strain. HosA-GFP fluorescence (green) indicates the localization of the fusion protein. Nuclei were stained with Hoechst 33,258 nucleic acid dye (blue). Scale bar, 10 μm. (C) Intensity profiles of HosA-GFP (green line) and Hoechst (blue line) signals were analyzed along the white arrow indicated in the merged images in (b) plotted against distance. (D) Western blot analysis of histone acetylation levels in wild-type, Δ*hosA* and three independent HosA-overexpression strains (OE:*hosA* #1, #2, #3). Membranes were probed with antibodies specific for acetylated histone H4K5/8/12/16, H4K5, H4K12, H4K16 and H3K18. Total Histone H4 and Histone H3 served as loading control. (E) Quantification of relative histone acetylation levels. Acetylation levels for each specific site were normalized to the total H4 signal. For the OE:*hosA* group, three independent transformants (OE:*hosA* #1, #2, and #3) were analyzed, and each point represents the mean of three biological replicates for one independent transformant. Data are presented as mean ± sd. Statistical significance was assessed using two-way ANOVA compared to the wild-type strain. **p < 0.01; ns, not significant.

To further investigate the molecular function of HosA, we assessed its subcellular localization by generating a C-terminal green fluorescent protein (GFP) fusion construct (HosA-GFP). Western blotting confirmed the successful expression of the fusion protein in *A. fumigatus*, with an observed molecular weight of approximately 81 kDa, consistent with the predicted sizes of HosA (~54 kDa) and GFP (~27 kDa) (Figure 3(A)). Fluorescence microscopy revealed that HosA-GFP green signals co-localized with the Hoechst-stained nuclei (Figure 3(B,C)), indicating that HosA predominantly localizes to the nucleus. This nuclear distribution aligns with its predicted role as a histone deacetylase. To further dissect the functional relevance of the conserved HDAC domain, we examined the subcellular localization of three catalytic-site point mutants (D133A, H175A, and D210A). The D133A and H175A variants retained exclusive nuclear localization, while the D210A mutant displayed partial cytoplasmic distribution, suggesting a potential defect in nuclear import or retention (Figure S8A). Given previous reports that the *S. cerevisiae* ortholog Hos2 targets acetylated lysine residues on histones^41^

We next evaluated the histone deacetylase activity of HosA in *A. fumigatus*. Western blot analysis using site-specific anti-acetyl-histone antibodies showed no significant difference in acetylation at H4K5/8/12/16 and H4K16 in the Δ*hosA* mutant compared to the wild-type strain, suggesting that loss of HosA does not markedly alter histone acetylation at these sites under the tested conditions (Figure 3(D,E)). The three point mutants (D133A, H175A, and D210A) exhibited similar acetylation patterns to the Δ*hosA* mutant (Figure S8B,C). However, overexpression of HosA in three independent transformants consistently resulted in a marked reduction in acetylation at histone sites H4K5, H4K16, and H4K5/8/12/16, confirming its deacetylase activity in *A. fumigatus*. In comparison, acetylation levels at H4K12 and H3K18 remained unaffected. Together, these results demonstrate that HosA acts as a functional histone deacetylase at the selected sites in *A. fumigatus*.

### Transcriptomic profiling reveals that HosA activates growth and developmental pathways while repressing secondary metabolite gene clusters

**Figure 4.**
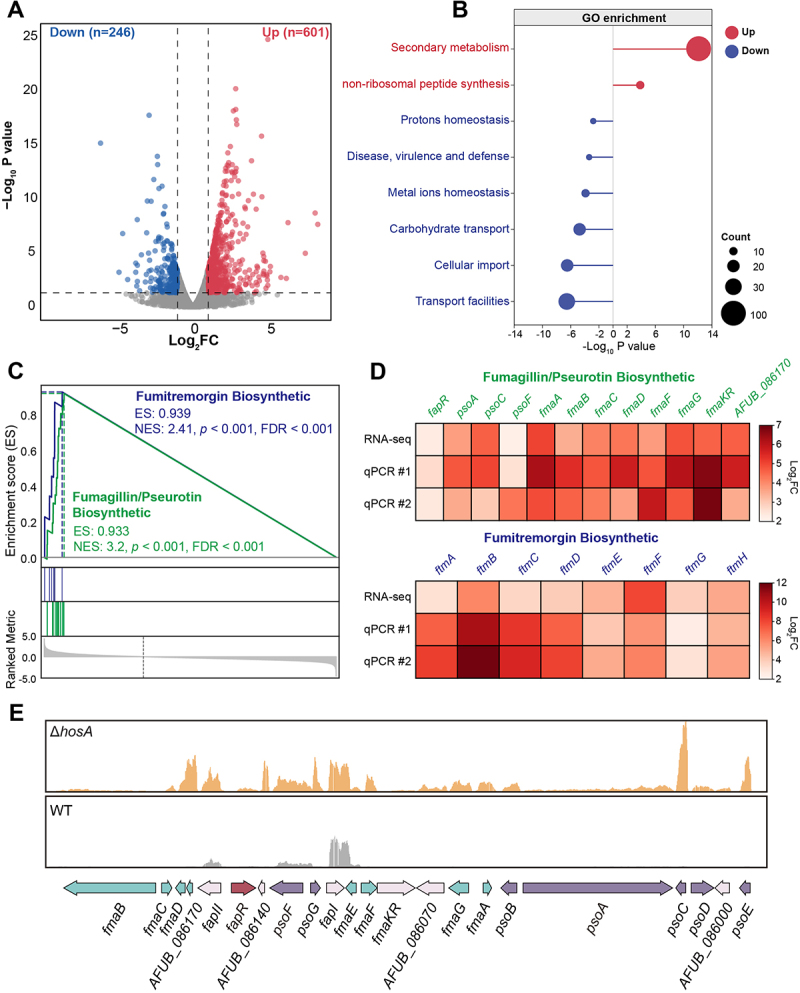
HosA represses secondary metabolite gene clusters while supporting developmental gene expression. (A) Volcano plot of global gene expression changes in the Δ*hosA* mutant. Red dots represent significantly upregulated genes, and blue dots represent significantly downregulated genes. Significance thresholds were set at log_2_ (fold change) ≥1.0 or ≤−1.0 and *p*-value <0.05. (B) Gene Ontology (GO) enrichment analysis of all differentially expressed genes between the wild-type and the Δ*hosA* mutant. (c) Gene Set Enrichment Analysis (GSEA) of secondary metabolite gene clusters. GSEA plots illustrate the enrichment of gene expression within the fumagillin/pseurotin biosynthetic supercluster and the fumitremorgin biosynthetic gene cluster. (D) Heatmap illustrating the differential expression of individual genes within the fumagillin/pseurotin biosynthetic supercluster and the fumitremorgin biosynthetic gene cluster. (e) Integrative Genomics Viewer (IGV) showing RNA-seq fragment counts for the Δ*hosA* mutant (orange peaks) and the wild-type strain (gray peaks) across a representative genomic region containing the fumagillin/pseurotin biosynthetic supercluster.

To explore the molecular basis underlying the pronounced phenotypes of the Δ*hosA* mutant including impaired growth and increased secondary metabolite production, we conducted transcriptomic profiling of the Δ*hosA* mutant compared to the wild-type strain. The transcriptomic analysis revealed that deletion of *hosA* led to genome-wide transcriptional changes, with 601 genes upregulated and 246 downregulated compared to the wild-type (Figure 4(A)). Gene Ontology (GO) enrichment analysis revealed that pathways related to secondary metabolism and non-ribosomal peptide synthesis were significantly up-regulated, whereas pathways important for growth and development were broadly down-regulated, including those associated with metal ion homeostasis, proton homeostasis, carbohydrate transport, and cellular import (Figure 4(B)), highlighting the critical role of HosA in controlling secondary metabolite biosynthesis and fungal development. To further examine this, we employed Gene Set Enrichment Analysis (GSEA) and revealed that genes within the fumitremorgin (Ftm) cluster and the fumagillin/pseurotin supercluster ranked among the most strongly upregulated in the Δ*hosA* mutant (Figure 4(C)). These transcriptomic findings were validated by RT-qPCR, which confirmed significantly elevated expression of these biosynthetic genes within both clusters, consistent with the RNA-seq data (Figure 4(D)). Supporting this result, visualization of RNA-seq reads using Integrative Genomics Viewer (IGV) confirmed the coordinated upregulation of adjacent genes within the fumagillin/pseurotin supercluster, further validating the elevated expression of this biosynthetic gene cluster in the absence of HosA (Figure 4(E)). The enhanced expression of the fumagillin/pseurotin supercluster is consistent with our earlier observation that the Δ*hosA* mutant produces elevated levels of fumagillin and pseurotin A (Figure 1(E,F)), further supporting a regulatory role of HosA in repressing this secondary metabolite pathway. Taken together, these results suggest that HosA acts as a negative regulator of secondary metabolism in *A. fumigatus*.

### HosA regulates secondary metabolism through locus-specific control of H4K16 acetylation

**Figure 5.**
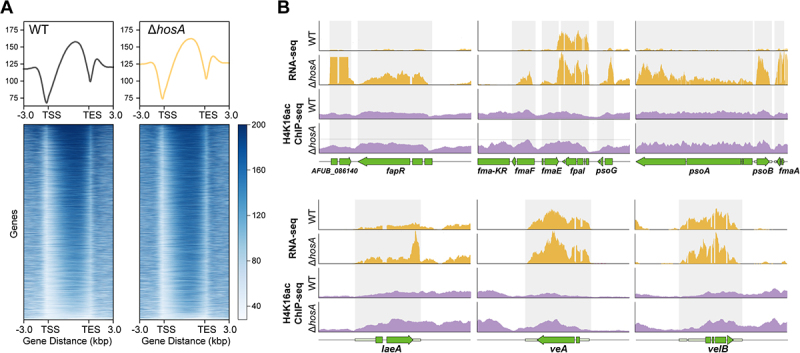
HosA modulates histone H4K16 acetylation at secondary metabolite gene clusters and velvet complex gene loci. (A) Genome-wide profiling of H4K16ac distribution in the wild-type and Δ*hosA* strains. Average profiles and heatmaps are shown relative to transcription start sites (TSS) and transcription end sites (TES) of all genes. (B) Genome browser views of H4K16ac ChIP-seq and RNA-seq profiles at representative genomic loci encompassing the fumagillin/pseurotin supercluster (*fapR*, *fmaF*, *fmaE*, *fpal*, *psoG*, *psoA*, *psoB* and *fmaA*) and the Velvet complex genes (*laeA*, *veA*, and *velB*).

Given its function as a histone deacetylase, we next examined whether the regulatory effects of HosA on secondary metabolism were mediated through changes in histone acetylation. To this end, we performed ChIP-seq profiling of histone H4K16 acetylation (H4K16ac) in the wild-type and Δ*hosA* strains. At the genome-wide level, H4K16ac in A. fumigatus was predominantly enriched over gene bodies, and deletion of *hosA* did not lead to substantial alterations in overall H4K16ac distribution (Figure 5(A)). This observation is consistent with our Western blot results showing no significant global differences in H4K16 acetylation between wild-type and *ΔhosA* strains (Figure 3(D,E)), indicating that HosA does not function as a global regulator of histone acetylation. In contrast, analysis of the fumagillin/pseurotin supercluster revealed clear locus-specific differences. In the Δ*hosA* mutant, H4K16ac enrichment was strongly increased across this cluster, consistent with the transcriptional upregulation observed in our RNA-seq data (Figure 5(B)). We also observed increased H4K16ac signals at the genomic regions corresponding to *laeA*, *veA*, and *velB*, which encode the core components of the Velvet complex. In line with the central role of this complex in coordinating fungal development and secondary metabolism, these findings indicate that HosA influences secondary metabolism through locus-specific regulation of histone H4K16 acetylation.

### Temperature-dependent variation in HosA expression determines fumagillin and pseurotin a biosynthesis

**Figure 6.**
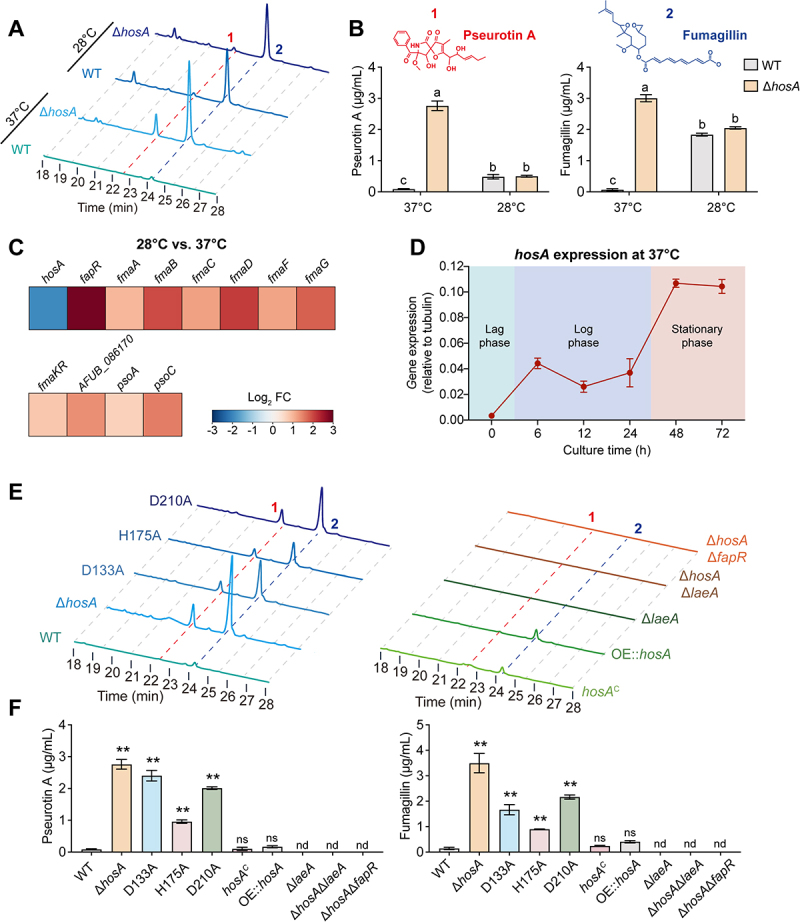
HosA is a negative regulator of secondary metabolite production in a temperature-dependent manner. (A) HPLC chromatograms of culture supernatants from wild-type (wt) strains and the Δ*hosA* mutant, following 72 h fermentation at either 28°C or 37°C in minimal medium. Peaks 1 and 2 correspond to pseurotin a and fumagillin, respectively. (B) Quantification of pseurotin a and fumagillin concentrations. Data represent the mean ± sd from three independent experiments. Different letters indicate significant differences (*p* < 0.01) as determined by one-way ANOVA. (C) Heatmap illustrating the relative expression levels of *hosA* and selected genes from the fumagillin/pseurotin supergene cluster in wild-type *A. fumigatus* grown at 28°C compared to 37°C.(D) Expression profile of *hosA* during different growth phases at 37°C. The relative expression of *hosA* (normalized to tubulin) was monitored by RT-qPCR at various time points during liquid culture at 37°C. Error bars represent standard deviation from three biological replicates. (E) HPLC chromatograms of culture supernatants from the indicated strains, following 72 h fermentation at 37°C in minimal medium. Peaks 1 and 2 correspond to pseurotin a and fumagillin, respectively.(F) Quantification of pseurotin a (left) and fumagillin (right) in the indicated strains. Data represent mean ± sd from three independent experiments. Statistical significance was determined by one-way ANOVA compared to the wild-type strain. **p < 0.01; ns, not significant; nd, not detected.

Since secondary metabolite production in filamentous fungi is often favored at lower temperatures (25–30°C), we assessed whether temperature influences HosA-mediated regulation of fumagillin and pseurotin A. To this end, we carried out the fermentation assay at the most common temperature 28°C. Consequently, under this condition, both fumagillin and pseurotin A were detected at comparable levels in the culture supernatants of wild-type and Δ*hosA* strains (Figure 6(A,B)), indicating that production of these metabolites occurs largely independently of HosA at 28°C. RT-qPCR analysis revealed that *hosA* expression was markedly lower at 28°C than at 37°C, while transcription of the fumagillin/pseurotin supercluster was elevated (Figure 6(C)), consistent with the reduced contribution of HosA to SM regulation at this lower temperature. Given our earlier observation that the wild-type strain does not produce fumagillin or pseurotin A at 37°C, whereas the Δ*hosA* mutant does, we examined whether differences in *hosA* expression might underlie this pattern. To test this, we monitored *hosA* expression throughout the growth cycle at 37°C. Transcript analysis revealed that *hosA* expression gradually increased during the logarithmic growth phase and remained consistently elevated as the culture entered the stationary phase (Figure 6(D)). Since secondary metabolism in filamentous fungi is typically activated during the stationary phase, the sustained high expression of *hosA* at this stage likely contributes to the continued repression of biosynthetic gene clusters.

To further evaluate whether this repression depends on the catalytic activity of HosA, we examined secondary metabolite production in strains carrying point mutations in HDAC domain. Notably, fumagillin and pseurotin A were detected in all three catalytic point mutants (D133A, H175A, D210A), but remained undetectable in the wild-type, complemented, and overexpression strains (Figure 6(E,F)). These findings suggest an essential role of the HDAC catalytic domain in mediating HosA-dependent repression of secondary metabolism. Furthermore, we deleted *fapR* or *laeA* (both known regulators of the fumagillin/pseurotin supercluster) on the Δ*hosA* background and confirmed correct strain construction by diagnostic PCR (Figure S9). Loss of either gene completely abolished fumagillin and pseurotin production, showing that the increased secondary metabolites in the Δ*hosA* mutant still required these two regulators. Taken together, our results demonstrate that HosA represses fumagillin and pseurotin biosynthesis via its HDAC domain by silencing the fumagillin/pseurotin supercluster.

### Loss of HosA derepresses fumagillin biosynthesis and enhances antagonism against Gram-positive bacteria

**Figure 7.**
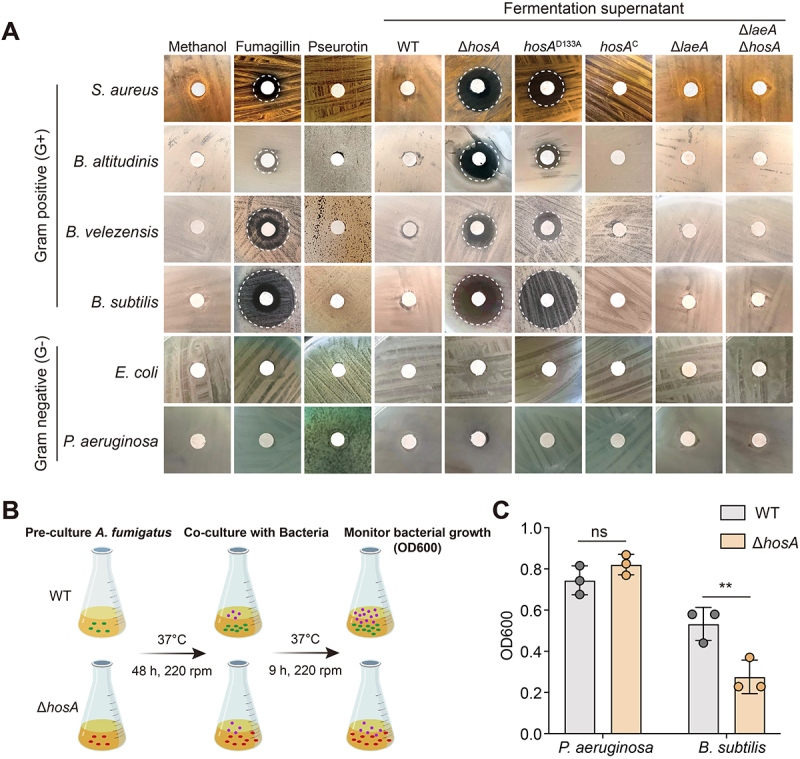
Crude extracts from *hosA* mutants exhibit antibacterial activity against Gram-positive bacteria. (A) Antibacterial activity of *A. fumigatus* culture supernatants derived from the indicated strains assessed by disk diffusion assay. Methanol served as a blank control. White dashed circles indicate zones of inhibition. (B) Schematic representation of the co-culture inhibition assay. Fungal strains were pre-cultured in MM at 37°C for 48 h, followed by 9 h coculture with bacteria. Bacterial growth was quantified by OD600 measurements. (C) Inhibition assays showing differential antibacterial effects of *A. fumigatus* wt and Δ*hosA* strains against Gram-negative *P. aeruginosa* and Gram-positive *B. subtilis*. Data represent the mean ± sd from three independent biological replicates. Statistical significance was assessed using unpaired *t*-test. **p < 0.01; ns, not significant.

Fungal secondary metabolites are broadly recognized as chemical defenses that protect against environmental competitors, including bacteria. Based on this concept, we hypothesized that fumagillin and pseurotin may contribute to the antagonistic interactions between *A. fumigatus* and neighboring bacterial species in its ecological niche. To evaluate this, agar diffusion assays were conducted to assess the antibacterial activity of fumagillin and pseurotin. The culture supernatant from the Δ*hosA* mutant, the *hosA* point mutant D133A, and commercial fumagillin standards, but not pseurotin, exhibited clear inhibitory effects against several Gram-positive bacterial species. In contrast, Gram-negative bacteria remained unaffected (Figure 7(A)). Furthermore, neither fumagillin nor pseurotin, whether as purified standards or crude fermentation extracts, showed any inhibitory activity against *A. fumigatus* itself (Figure S10). These results suggest that fumagillin may serve as a selective defense molecule produced by *A. fumigatus* that specifically targets Gram-positive bacteria. Consistent with these findings, co-culture inhibition assays further showed that after pre-culturing *A. fumigatus* for 24 h to allow secondary metabolite production to initiate, followed by co-culturing with bacteria for 9 h prior to OD measurement, Δ*hosA* displayed strong inhibition of *B. subtilis*, whereas the wild-type strain did not exhibit detectable antagonism under the same conditions, and neither strain inhibited the Gram-negative bacteria *P. aeruginosa* (Figure 7(B,C)). Together, these results demonstrate that loss of HosA derepresses fumagillin biosynthesis and results in enhanced antagonistic activity of *A. fumigatus* specifically against Gram-positive bacteria.

## Discussion

**Figure 8.**
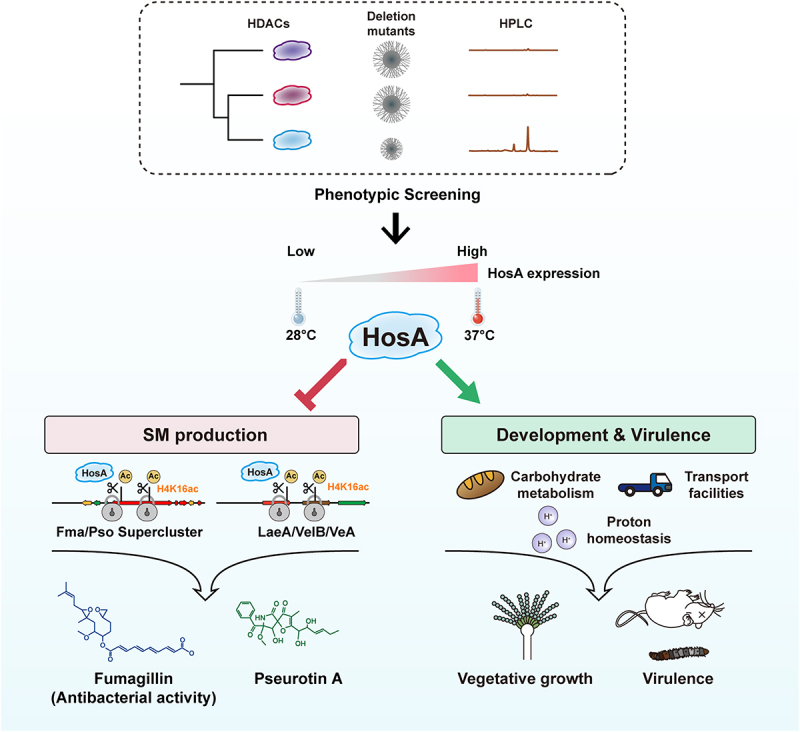
A proposed model for HosA-mediated regulation of *A. fumigatus* development, virulence and secondary metabolism.

To thrive in diverse and competitive settings both within the host and in external environments, fungal pathogens must coordinate multiple physiological processes such as hyphal growth, conidiation and metabolism to adapt to fluctuating conditions. Among these, a critical trade-off exists between vegetative development and secondary metabolism, which compete for metabolic resources and serve distinct ecological functions. Understanding the molecular mechanisms that balance this trade-off is essential for elucidating fungal pathogenicity and identifying potential antifungal targets. In this study, we identify the class I histone deacetylase HosA as a dual-function regulator in *A. fumigatus* that promotes vegetative growth and virulence while repressing secondary metabolism through direct chromatin-level control of biosynthetic gene clusters and modulation of upstream developmental regulators within the Velvet complex (Figure 8). We further demonstrate that *hosA* expression varies with temperature, suggesting that HosA contributes to adjusting developmental and metabolic priorities under different environmental conditions. Together, these findings support a model in which HosA coordinates fungal growth and chemical defense to optimize fitness across fluctuating environments.

Although structurally conserved across eukaryotes, Hos2 family histone deacetylases are associated with diverse phenotypic outcomes across fungal species. In *S. cerevisiae*, Hos2 participates in transcriptional elongation via the Set3 complex [29,41] while in *Candida albicans*, it influences morphogenesis through interactions with the cAMP-PKA pathway [42]. In *Ustilago maydis*, Hos2 promotes the yeast-to-hyphal transition by activating mating-type genes and contributes to virulence [43]. In filamentous fungi, HosA in *Aspergillus niger* contributes to pigment biosynthesis, sporulation, and stress resistance [44]. Its deletion enhances kojic acid production, whereas in *Aspergillus flavus*, HosA represses aflatoxin biosynthesis and modulates oxidative stress responses [45]. Our findings revealed a bifunctional role of HosA in *A. fumigatus* that coordinates growth, virulence, and secondary metabolism. Overexpression of *hosA* enhances colony growth compared to the wild-type (Figure S4), highlighting its role in supporting vegetative growth, whereas deletion of *hosA* derepressed secondary metabolite biosynthesis. These results underscore the evolutionary plasticity of Hos2 orthologs and suggest that their functions have adapted to meet species-specific regulatory and physiological demands.

At the mechanistic level, our data suggest that HosA regulates secondary metabolism through chromatin-based control of histone acetylation. At the genome-wide scale, H4K16ac in *A. fumigatus* displayed a distribution pattern predominantly enriched over gene bodies, consistent with observations in other species [46,47]. H4K16ac ChIP-seq revealed clear locus-specific increases in acetylation across the fumagillin/ pseurotin supercluster in the Δ*hosA* mutant, consistent with the elevated transcription of these biosynthetic genes. In addition to its direct effects on secondary metabolite gene clusters, we observed increased H4K16ac signals at the genomic regions encoding LaeA, VeA, and VelB, three core components of the Velvet complex, in the Δ*hosA* mutant (Figure 5). These results suggest that HosA may indirectly modulate secondary metabolism by altering the chromatin environment of these global regulatory genes. Given that the Velvet complex is a well-established mediator of light-responsive developmental and metabolic regulation in fungi [17,18], it will be interesting to determine whether HosA-dependent chromatin regulation is linked to light-responsive pathways. However, as our experiments were not performed under controlled light conditions, the contribution of light to HosA function remains unresolved. Likewise, because the H4K16ac ChIP-seq analysis was performed at a single 24 h time point, our data capture the chromatin state associated with HosA-dependent regulation at this stage, but not its temporal dynamics. In addition to HosA, the sirtuin family HDAC SirE also contributes to the epigenetic control of secondary metabolism. HPLC profiling showed that deletion of *sirE* led to elevated fumagillin and pseurotin A production in *A. fumigatus* (Figure 1). These results indicate that HosA and SirE may operate to fine-tune fungal metabolism. Consistent with our findings, recent work shows that sirtuin family HDACs also contributes to secondary metabolism, stress response, and virulence in *A. fumigatus* [48]. Although deletion of other HDAC genes did not result in obvious phenotypes under our test conditions, they may still have roles under different environmental or host-related conditions that were not examined in our study. Nevertheless, the regulatory role of HosA extends beyond secondary metabolism, HosA also appears to positively influence core metabolic processes and fungal virulence, as deletion of hosA led to downregulation of genes involved in carbohydrate transport, metal ion homeostasis, and proton homeostasis (Figure 4(B)), all of which are important for fungal growth and development. Moreover, the loss of HosA significantly impairs fungal virulence in a *G. mellonella* and mouse infection model (Figure 2 and Figure S7), suggesting that its activity is critical for pathogenicity. It is also worth noting that the Δ*hosA* strain shows marked growth and developmental defects *in vitro*, which may contribute to the reduced virulence phenotype. Together, these findings underscore HosA as a dual-function regulator in *A. fumigatus*, acting as a repressor of secondary metabolism and a positive regulator of metabolic fitness and virulence.

Interestingly, our findings revealed that the balance between developmental and metabolic outputs controlled by HosA appears to be influenced by temperature-associated changes in *hosA* expression. It has been observed that the expression of many biosynthetic gene clusters (BGCs) becomes silenced under standard lab or unstimulated conditions, temperature is one of the environmental factors that can shift SM biosynthesis [49]. In our study, *hosA* expression is markedly lower at 28°C than at 37°C, whereas transcription of the fumagillin/pseurotin supercluster increases, consistent with enhanced metabolite production. At 37°C, *hosA* expression remains high and these clusters stay repressed, coinciding with stronger vegetative growth (Figure 6). These results suggest that HosA may help *A. fumigatus* adjust metabolic priorities under different growth conditions. Accordingly, our data support a model in which HosA links chromatin-based control of secondary metabolism with the regulation of fungal development. By modulating metabolite biosynthesis and developmental programs in a condition-dependent manner, HosA enables *A. fumigatus* to balance pathogenicity, growth, and ecological competitiveness. These findings advance our understanding of fungal environmental adaptation and point to new opportunities for antifungal strategy development and secondary metabolite engineering.

## Materials and methods

### Strains, media, and culture conditions

All *A. fumigatus* strains used in this study are listed in Table S1. Unless otherwise stated, strains were cultured at 37°C under standard laboratory conditions. Minimal medium (MM) consisted of 10 g/L glucose, 1 mL/L trace element solution, and 50 mL/L of a 20 × salt stock. For solid media, 2% (w/v) agar was added. The final pH was adjusted to 6.5. Yeast-glucose (YG) medium contains 20 g/L glucose, 5 g/L yeast extract, and 1 mL/L trace element solution. For solid YG medium, 2% (w/v) agar was added to the base YG formulation. Bacterial strains used in the study included *Staphylococcus aureus*, *Bacillus altitudinis*, *Bacillus velezensis*, *Bacillus subtilis*, *Escherichia coli,* and *Pseudomonas aeruginosa* and were kindly provided by the laboratories of Prof. Bin Lian and Prof. Chuanchao Dai at Nanjing Normal University, China. All bacterial strains were cultivated in Luria–Bertani (LB) medium at 37°C under aerobic conditions unless otherwise specified.

### Construction of strains

Primer sequences used in this study are listed in Table S2. The *hosA* deletion construct was generated by replacing the entire open reading frame with the hygromycin resistance marker (*hph*). Upstream and downstream flanking regions were amplified from the Δ*ku80* strain using primer pairs HosA-P1/P3 and HosA-P4/P6, respectively. The hph cassette was amplified from plasmid pAN7-1 using primers hph-full-F/R. The three fragments were fused by overlap PCR with primers HosA-P2/P5 to generate the deletion cassette, which was introduced into Δ*ku80* via PEG-mediated protoplast transformation as previously described[50]. Transformants were selected on 200 μg/mL hygromycin and verified by diagnostic PCR using HosA-P1/hph-R and HosA-self-F/R (Figure S1). Other single-gene knockout strains were constructed following the same strategy. For double knockouts, a phleomycin resistance marker was used, and transformants were selected on 200 μg/mL hygromycin B and 25 μg/mL bleomycin sulfate.

For genetic complementation, the full-length *hosA* gene including its native promoter, 5’UTR, ORF, and 3’UTR was amplified from wild-type genomic DNA using primers HosA-phle-F/R, fused to the phleomycin resistance marker, and cloned into pSinoMol Zero Blunt. After amplification in *E. coli* DH5α, the construct was introduced into the Δ*hosA* strain for random genomic integration. Transformants were confirmed by PCR. For heterologous complementation, the plasmid backbone was linearized with primers Linear-HosA-F/R, and the *S. cerevisiae* homolog ScHOS2 was amplified from cDNA using primers ScHos2-F/R. The fragments were assembled into a plasmid containing the phleomycin resistance cassette and transformed into the Δ*hosA* strain.

To generate the HosA-GFP strain, upstream and downstream regions flanking the *hosA* stop codon (excluding the stop codon) were amplified using primer pairs HosA-GFP-P1/P3 and HosA-GFP-P4/P6. A GFP-pyrG cassette was amplified from plasmid pFNO3. The three fragments were fused by overlap PCR and cloned into pSinoMol Zero Blunt, then transformed into A1160. Transformants were verified by diagnostic PCR and Western blotting. The HosA-FLAG strain was constructed using the same strategy with a hygromycin resistance marker.

Site-directed mutagenesis was performed using the HosA-GFP plasmid as template. Mutated fragments were amplified with mutation-specific primer pairs and circularized using the Mut Express II Fast Mutagenesis Kit (Vazyme). Constructs were verified by sequencing, amplified in *E. coli* DH5α, and transformed into A1160. The *hosA* HDAC domain in transformants was sequenced to confirm the presence of the desired mutations.

### Fluorescence microscopy

To visualize the subcellular localization of the HosA-GFP fusion protein, conidia (1 × 10^6^) were inoculated into 1 mL of liquid minimal medium (MM) and transferred onto a sterile coverslip. The cultures were incubated at 37°C for 14 h to allow germination. After incubation, the coverslips were gently washed with phosphate-buffered saline (PBS) to remove excess mycelia, and the cells were fixed with 4% paraformaldehyde (Sangon Biotech, E672002) at room temperature for 40 min. Following fixation, nuclei were stained with Hoechst 33,258 (Sangon Biotech, E607329) at a final concentration of 2 μg/mL for 5 min, then washed thoroughly with PBS to remove the excess dye. The coverslip was mounted onto a microscope slide and examined using a fluorescence microscope (Olympus IX71). Images were captured using a Prime sCMOS camera (Teledyne Photometrics) and processed using ImageJ software.

### Virulence assay

Virulence was assessed using both *G. mellonella* larvae and a murine infection model. For the *G. mellonella* assay, spore suspensions were prepared at 1 × 10^8^ conidia/mL in phosphate-buffered saline (PBS). A 10 μL aliquot of the suspension was injected into the left proleg of each larva using a manual microsyringe. Control larvae received 10 μL of sterile PBS. Infected larvae were incubated at 37°C and survival was monitored daily.

For the murine model, the infection procedure was conducted according to a previously described protocol [51], with minor modifications. Eight-week-old female C57BL/6 mice (Vital River Laboratory Animal Technology, Zhejiang, China) were immunosuppressed via intraperitoneal injection of cyclophosphamide (150 mg/kg) on days −4 and −1 prior to inoculation. Conidial suspensions were adjusted to 1.5 × 10^8^ conidia/mL using a hemocytometer. Mice were anesthetized with isoflurane for anesthesia, and 30 μL of the conidial suspension was administered intratracheally by gently extending the tongue to open the airway and allowing the inoculum to enter the lungs. Mouse body weights were monitored daily, and animals were humanely euthanized under deep isoflurane anesthesia if they experienced a ≥20% loss from their initial weight. For survival analysis, eight mice per group were monitored twice daily over a 10-day period to evaluate disease progression and mortality. Survival data were analyzed using the log-rank (Mantel-Cox) test. For histopathological analysis, mice were humanely euthanized under deep isoflurane anesthesia 72 h post-infection, and lung tissues were collected and processed for Grocott’s methenamine silver (GMS) and hematoxylin and eosin (H&E) staining according to established protocols.

For fungal burden analysis, three mice per group were euthanized by cervical dislocation at 72 h post-infection. The lungs were aseptically removed, weighed, and homogenized in 5 mL sterile phosphate-buffered saline (PBS) using a tissue homogenizer. Homogenates were serially diluted 10-fold in sterile PBS, and 100 μL of each dilution was plated onto solid YG medium containing 150 μg/mL ampicillin to suppress bacterial growth. Plates were incubated at 37°C for 10 h, and colonies were counted. Fungal burden was calculated as colony-forming units (CFU) per gram of lung tissue.

### Ethics statement

All animal procedures were approved by the Institutional Animal Care and Use Committee (IACUC) of Nanjing Normal University (approval number: IACUC-20230231) and were performed in accordance with the ARRIVE guidelines. All animal experiments, including anesthesia and euthanasia procedures, were conducted in accordance with the American Veterinary Medical Association (AVMA) guidelines. All RNA-seq and ChIP-seq data in this manuscript were generated by the authors in this study and subsequently deposited by the authors into public repositories (NCBI SRA and NGDC GSA) for data sharing purposes. The use of these public repositories does not require additional ethical approval or permission from data owners, as the deposited datasets do not contain personal, clinical, or identifiable information.

### Quantitative real-time PCR and RNA-sequencing

Total RNA was extracted from fungal mycelia using the RNA Purification Kit (Sangon Biotech, B511361-0100) according to the manufacturer’s instructions. A total of 500 ng RNA was reverse-transcribed into cDNA using the HiScript III Reverse Transcription Kit (Vazyme, R323-01). The resulting cDNA was diluted 1:5 with nuclease-free water and used as the template for quantitative real-time PCR (qRT-PCR). qRT-PCR was performed using SYBR qPCR Master Mix (Vazyme, Q331) on a QuantStudio 3 Real-Time PCR System (Thermo Fisher Scientific). Three technical replicates were included for each target gene. Gene expression levels were normalized to the internal reference gene tubulin, and relative expression was calculated using the 2^−ΔΔCt^ method. RNA sequencing (RNA-seq) was carried out on the Illumina platform following standard protocols by Personalbio (Shanghai, China). Three independent biological replicates were generated for each strain.

### Western blotting analysis

Mycelia cultured for 24 h were harvested, flash-frozen in liquid nitrogen, and ground into a fine powder using a pre-chilled mortar and pestle. Total protein was extracted as previously described [52]. Protein samples were separated via SDS-PAGE (GenScript, M00930) and transferred onto PVDF membranes (Merck Millipore, IPVH00010) using the Fast Wet Transfer System (GenScript, eBlo^TM^ L1). The following primary antibodies were used: anti-FLAG (1:5000; Abclonal, AE092), anti-β-Actin (1:50000; Abclonal, AC026), anti-GFP (1:2000; Roche, 11814460001), anti-H4 (1:2000; PTM Bio, PTM-1015), anti-H3K18ac (1:500; Abclonal, A7257), anti-H4K5ac (1:2000; PTM Bio, PTM-163), anti-H4K12ac (1:2000; PTM Bio, PTM-165), anti-H4K16ac (1:2000; PTM Bio, PTM-122), and anti-H4K5/8/12/16ac (1:2000; PTM Bio, PTM-189). HRP-conjugated goat anti-mouse (1:5000; Abclonal, AS003) and goat anti-rabbit (1:5000; Abclonal, AS014) antibodies were used as secondary antibodies. Signal detection was performed using an enhanced chemiluminescence (ECL) kit (Vazyme, E411). Images were captured using a Tanon 4200 imaging system, and band intensities were quantified using ImageJ software.

### Chromatin immunoprecipitation sequencing (ChIP-seq)

Chromatin immunoprecipitation followed by sequencing (ChIP-seq) was performed using an anti-H4K16ac antibody (PTM Bio, PTM-122) in both wild-type and Δ*hosA* strains, with two biological replicates prepared for each strain. A total of 10^8^ spores were inoculated into 100 mL liquid minimal medium (MM) and cultured at 37°C for 24 h. This time point was selected based on transcriptomic data showing that transcriptional differences between the wild-type and Δ*hosA* strains were already established at this stage. Mycelia were collected by filtration, washed with ice-cold PBS, flash-frozen in liquid nitrogen, and shipped on dry ice to Igenebook (Wuhan, China), where the samples were subjected to crosslinking, chromatin immunoprecipitation, library construction, and next-generation sequencing. ChIP-seq signal profiles were visualized and analyzed using IGV (version 2.18.2) and deepTools (version 3.5.5).

### Secondary metabolite extraction and analysis

For secondary metabolite extraction, *A. fumigatus* conidia (1 × 10^8^) were inoculated into 100 mL of minimal medium (MM) and incubated at 28°C or 37°C with shaking at 220 rpm for 3 days. Unless otherwise specified, cultures were incubated under ambient laboratory light (scattered room light) without specific light control. After cultivation, culture supernatants were collected and lyophilized. The resulting powder was accurately weighed and re-dissolved in 2 mL of methanol with ultrasonic treatment for 3 h. The extract was subsequently used for high-performance liquid chromatography (HPLC) and liquid chromatography–mass spectrometry (LC-MS) analysis.

HPLC was performed using a 1260 Infinity II LC System (Agilent Technologies) equipped with a Welch UltimateⓇ AQ-C18 column (4.6 × 250 mm, 5 μm). The mobile phase consisted of 0.05% formic acid in water (solvent A) and acetonitrile (solvent B). The flow rate was set at 1 mL/min. The gradient program was as follows: 75% A to 35% A over 20 min, held at 35% A for 8 min, and then returned to 75% A within 7 min, for a total run time of 35 min. The injection volume was 20 μL, and the column temperature was maintained at 20°C. Detection was performed using a Multi-Wavelength Detector (MWD) at 220, 270 and 300 nm (bandwidth: 4 nm).

LC-MS was conducted at the Analytical and Testing Center of Nanjing Normal University using an Agilent LC1290/MS6470B system. Chromatographic conditions (column, solvents) matched those used for HPLC, except the flow rate was adjusted to 0.6 mL/min. The gradient was as follows: 75% A to 35% A over 20 min, held at 35% A for 20 min, returned to 75% A in 1 min, and held at 75% A for 4 min (total run time: 45 min). Samples (10 μL) were injected without temperature control. Mass detection was performed in positive ion mode using an Agilent Jet Stream (AJS) electrospray ionization (ESI) source. The system was set to scan ions with m/z ranging from 100 to 1000. The ion source was operated at 300°C with a gas flow of 5 L/min and a nebulizer pressure of 40 psi.

### Diffusion-based bioactivity assays

The antibacterial activity of fungal metabolites was assessed using a disk diffusion assay. Briefly, 100 μL of an overnight bacterial culture (OD_600_ = 1.0) was spread onto the surface of freshly prepared LB agar plates using a sterile cotton swab. After allowing the surface to dry for 10 min, sterile filter paper discs (8 mm in diameter) were placed onto the agar surface. Each disc was loaded with 10 μL of either the fungal extract obtained from HPLC preparation or methanol (solvent control). The plates were incubated at 37°C for 24 h. Following incubation, zones of inhibition were measured to evaluate antibacterial activity.

To assess potential self-inhibitory activity against *A. fumigatus*, an Oxford cup diffusion assay was performed. Briefly, a total of 10^7^ *A. fumigatus* spores were evenly spread onto the surface of solid medium using a sterile cell spreader. Sterile stainless-steel Oxford cups (7.8 mm in diameter) were placed on the agar surface and filled with 200 μL of one of the following: methanol as a negative control, purified fumagillin (250 μg/mL in methanol), purified pseurotin A (100 μg/mL in methanol), 50-fold concentrated wild-type culture supernatant, or 50-fold concentrated Δ*hosA* culture supernatant. Plates were incubated at 37°C for 24 h and then examined for zones of growth inhibition around the cups.

### Bioinformatic analysis

HosA ortholog protein sequences used were retrieved from the NCBI database and aligned using MUSCLE with default parameters. Phylogenetic trees were constructed in MEGA11 using the neighbor-joining method with 1000 bootstrap replicates. Domain architectures were predicted using the SMART database. For analysis of conserved residues within the HDAC domain, protein sequences from *A. fumigatus*, *Saccharomyces cerevisiae* (Hos2), and *Homo sapiens* (HDAC1) were aligned using ClustalW with default parameters in MEGA11. The alignment was visualized and annotated using Jalview version 2.11.

### Statistical analysis

All statistical analyses were performed using GraphPad Prism version 9.0 (GraphPad Software, USA). Comparisons between two groups were conducted using Student’s *t*-test, while comparisons among three or more groups were performed using one-way or two-way analysis of variance (ANOVA) as appropriate. Survival data were analyzed using the log-rank (Mantel-Cox) test. Data are presented as mean ± standard deviation (SD) from at least three independent biological replicates.

## Supplementary Material

QVIR-2025-1195.R1- Clean copy of supplementary figure and table legends.docx

FigS2.tif

FigS3.tif

FigS6.jpg

FigS9.tif

FigS8.tif

FigS5.tif

FigS10.tif

20260317_Supplementary TableS2.docx

FigS1.jpg

FigS7.tif

FigS4.tif

20260317_Supplementary TableS1.docx

## Data Availability

The RNA-seq data generated in this study have been deposited in the NCBI Sequence Read Archive (SRA; https://www.ncbi.nlm.nih.gov/sra) under accession number PRJNA1030787. The raw H4K16ac ChIP-seq data have been deposited in the Genome Sequence Archive (GSA; https://ngdc.cncb.ac.cn/gsa) at the National Genomics Data Center, China National Center for Bioinformation/Beijing Institute of Genomics, Chinese Academy of Sciences, under accession number CRA035305. All supplementary figures and tables, all raw numerical data underlying the figures, together with the corresponding statistical calculation files, are available via Figshare (https://doi.org/10.6084/m9.figshare.30993061) [53]. Original, unprocessed microscopy images, immunofluorescence images, and uncropped blot and gel images corresponding to the figures presented in this manuscript are also deposited in the same Figshare repository.
