# Supplementary material for: HosA-mediated epigenetic regulation of growth, virulence, and secondary metabolism in Aspergillus fumigatus
Source: Virulence. 2026 Apr 2;17(1):2655064. doi: 10.1080/21505594.2026.2655064 (PMC13078215; doi:10.1080/21505594.2026.2655064)
Supplement: QVIR-2025-1195.R1- Clean copy of supplementary figure and table legends.docx [file KVIR_A_2655064_SM2071.docx]

**Supplementary Figure and Table Legends**

**Figure S1. Construction and diagnostic PCR confirmation of HDAC gene deletion mutants in *A. fumigatus*.**

**(A)** Schematic representation of the gene deletion strategy for *A. fumigatus* HDACs. Diagram illustrates the homologous recombination approach used to generate deletion mutants.

**(B)** Diagnostic PCR confirmation of individual HDAC gene deletion mutants.

**(C)** Schematic overview of *hosA* complementation (*AfhosA* or *Schos2*) and diagnostic PCR validation of the resulting complementation mutants.

**Figure S2. LC-MS identification of fumagillin and pseurotin A from Δ*hosA* culture supernatant.**

The HPLC chromatogram (top panel) of the Δ*hosA* mutant culture supernatant displays two prominent peaks (at 26.460 min and 29.153 min) corresponding to secondary metabolites. Subsequent LC-MS/MS analysis (bottom panels) identified the peak at 26.460 min as pseurotin A ([M+H]^+^ m/z 433.2) and the peak at 29.153 min as fumagillin ([M+H]^+^ m/z 459.2).

**Figure S3. Evolutionary conservation and domain architecture of HosA orthologs across diverse fungal species.**

Phylogenetic tree of *A. fumigatus* HosA and orthologs from selected fungal species, constructed using the neighbor-joining method based on amino acid sequences (Left panel). Predicted domain architectures of HosA and representative orthologs, highlighting the conserved HDAC domain (Right panel).

**Figure S4. Overexpression of *hosA* promotes growth in *A. fumigatus.***

**(A)** Colony morphology of the wild-type (WT), Δ*hosA* mutant and three independent *hosA* overexpression strains (OE::*hosA* #1, OE::*hosA* #2, OE::*hosA* #3) grown on solid minimal medium for 48 h at 37°C.

**(B)** Relative expression level of *hosA* in the indicated strains normalized to the wild type. Data are presented as mean ± SD from three independent experiments. Statistical significance was determined by one-way ANOVA compared to the wild-type strain. **, *p* < 0.01.

**(C)** Quantification of colony diameters for the indicated strains. Data are presented as mean ± SD from three independent experiments. Statistical significance was determined by one-way ANOVA compared to the wild-type strain. **, *p* < 0.01; *, *p* < 0.05; **ns, not significant.**

**Figure S5. Sequence analysis and expression verification of HosA point-mutation variants.**

**(A)** Sequence alignment of HosA orthologs highlighting conserved residues. Partial protein sequence alignment of HosA orthologs from *A. fumigatus*, *S. cerevisiae* and *H. sapiens*. Conserved amino acid residues within the HDAC domain are indicated with the 133rd, 175th and 210th positions specifically highlighted as sites targeted for point mutations.

**(B)** Western blot analysis using an anti-GFP antibody confirms the correct expression of HosA-GFP (wild-type) and its point-mutation fusion proteins in *A. fumigatus*. Coomassie Brilliant Blue (CBB) staining was used as a loading control.

**Figure S6. HosA is involved in the *A. fumigatus* response to various environmental stressors.**

**(A)** Phenotypic characterization of *A. fumigatus* *hosA* mutants under various stress conditions. Wild-type, Δ*hosA*, *hosA* point-mutation strains and *hosA* complementation strain were inoculated onto solid minimal medium (MM) and incubated for 48 h under the indicated stress conditions or at different temperatures. Stress-inducing agents included 10 mM HU (hydroxyurea), 2 mM H_2_O_2_, 20 μM CPT (camptothecin), 5 mM ferrous chloride, 10 mM manganese chloride, 200 mM magnesium chloride, 2 μM vitamin K, and 18 μg/mL Congo red.

**(B)** Quantitative analysis of inhibition rates for the indicated strains under stress. A heatmap displays the percentage inhibition rate for each strain under the indicated stress conditions, relative to their respective growth on control medium. Inhibition rate is calculated as the reduction in colony diameter. Asterisks (**) indicate significant differences (*p* < 0.01) compared to the wild-type.

**Figure S7. Loss of HosA significantly reduces fungal burden in murine lungs.**

**Quantification of fungal burden (CFU counts) in mouse lung homogenates 3 days post-infection. Data represent the mean ± SD from at least three mice per group. Statistical analysis was performed using one-way ANOVA. **, *p* < 0.01; ns, not significant.**

**Figure S8. Subcellular localization and histone acetylation analysis of HosA point-mutation variants.**

**(A)** Subcellular localization of GFP-tagged HosA point mutants. Nuclei were stained with Hoechst 33258 nucleic acid dye. Representative images from three independent experiments are shown. Scale bars, 10 µm.

**(B)** Western blot analysis of histone acetylation in point-mutation strains. Membranes were probed with antibodies specific for acetylated histone H4K5/8/12/16, H4K16 and H3K18. Total Histone H4 and Histone H3 served as loading controls.

**(C)** Quantification of relative histone acetylation levels in point-mutation strains. Acetylation levels were normalized to the total Histone H4 signal. Data represent the mean ± SD from three independent biological replicates. Statistical analysis was performed using one-way ANOVA. ns, not significant.

**Figure S9. Construction and diagnostic PCR confirmation of *laeA* and *fapR* gene deletion mutants.**

**(A)** Schematic overview of the homologous recombination strategy used to construct *laeA* and *fapR* deletion mutants in *A. fumigatus*.

**(B)** Diagnostic PCR confirmation of *laeA* gene deletion mutants.

**(C)** Diagnostic PCR verification of *laeA* and *fapR* deletions generated on the Δ*hosA* background.

**Figure S10. Oxford cup diffusion assays evaluating the potential self-inhibitory effects of fumagillin, pseurotin A and fungal culture supernatants on *A. fumigatus*.**

Oxford cup diffusion assays were performed on agar plates seeded with 10^7^ *A. fumigatus* spores. Oxford cups were filled with 200 μL methanol, purified fumagillin (250 μg/mL), purified pseurotin A (100 μg/mL), or 50-fold concentrated culture supernatants from the wild-type or Δ*hosA* strains. Plates were incubated at 37°C for 24 h.

**Table S1. Strains used in this study.**

**Table S2. Primers used in this study.**
