# Supplementary figures and images for: HosA-mediated epigenetic regulation of growth, virulence, and secondary metabolism in Aspergillus fumigatus
Source: Virulence. 2026 Apr 2;17(1):2655064. doi: 10.1080/21505594.2026.2655064 (PMC13078215; doi:10.1080/21505594.2026.2655064)

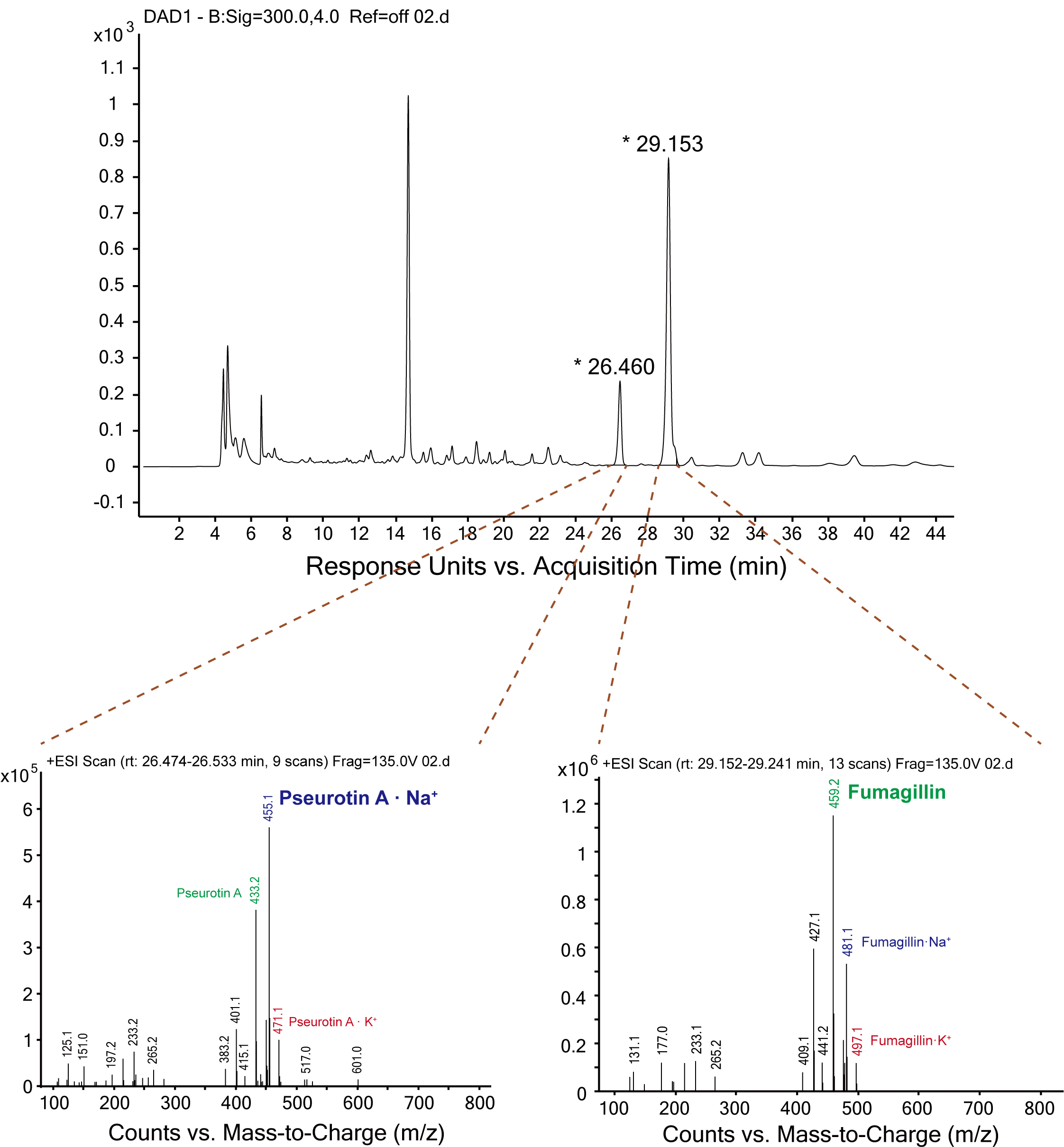

Supplement: FigS2.tif [file KVIR_A_2655064_SM2070.tif]

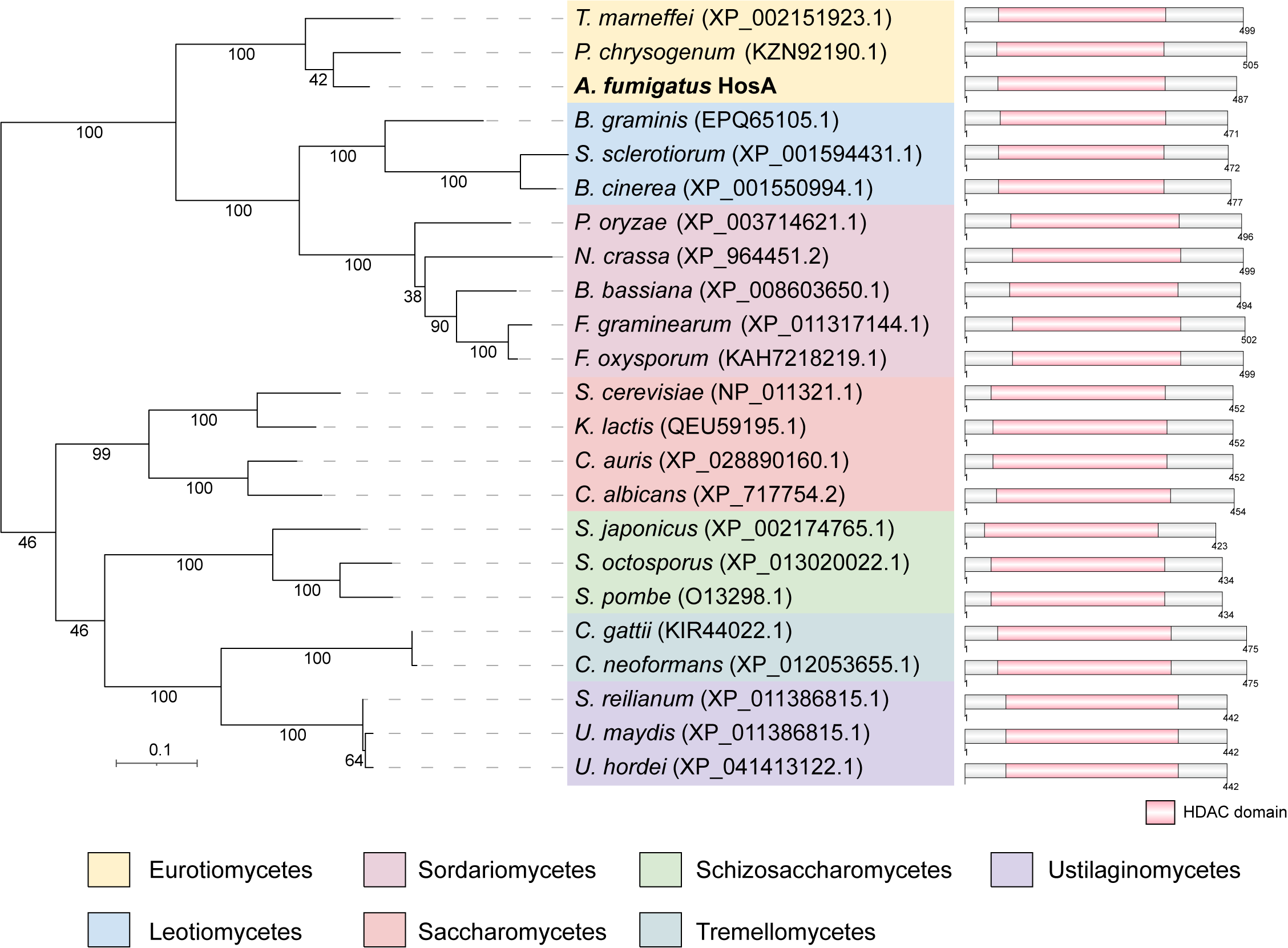

Supplement: FigS3.tif [file KVIR_A_2655064_SM2069.tif]

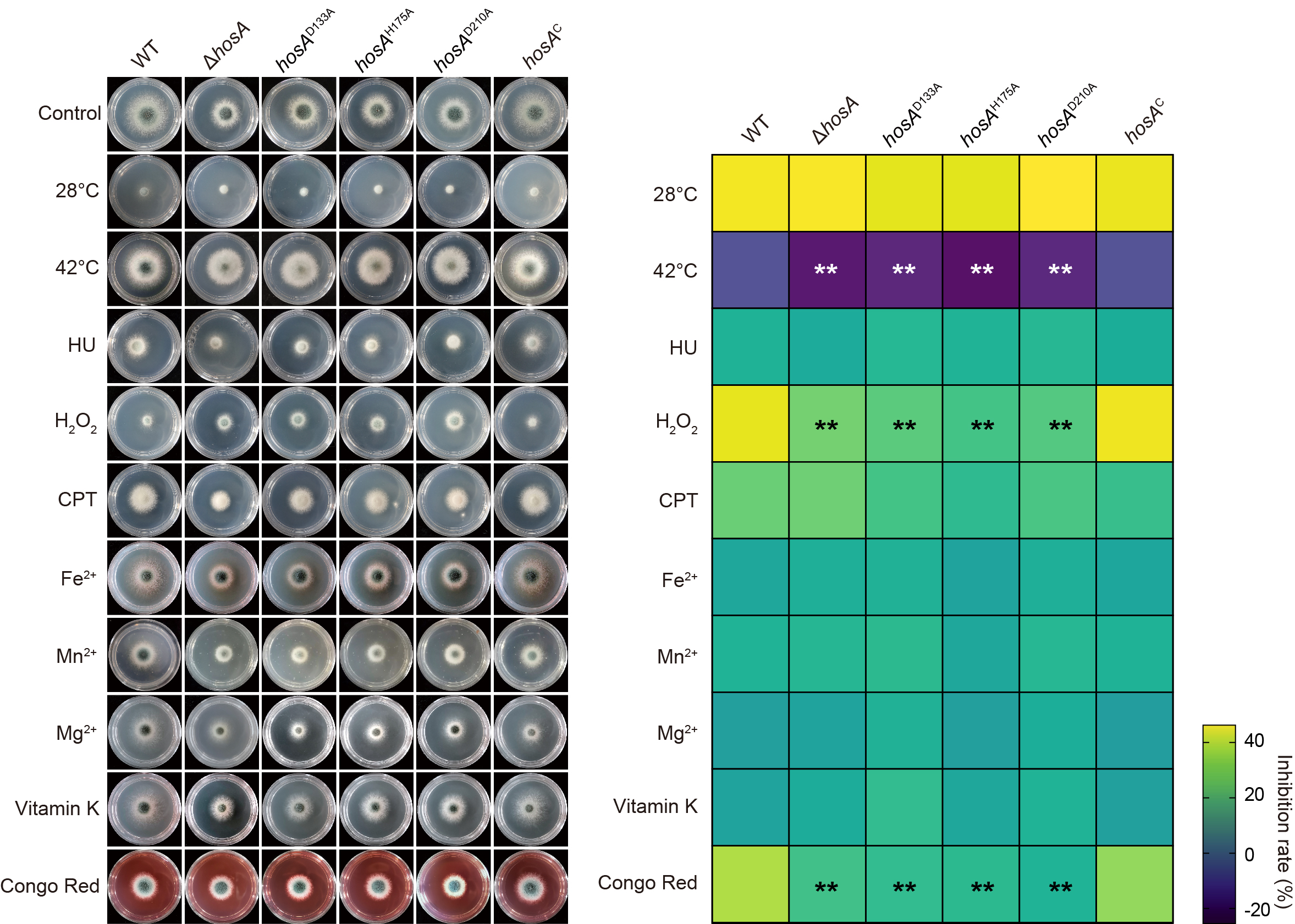

Supplement: FigS6.jpg [file KVIR_A_2655064_SM2068.jpg]

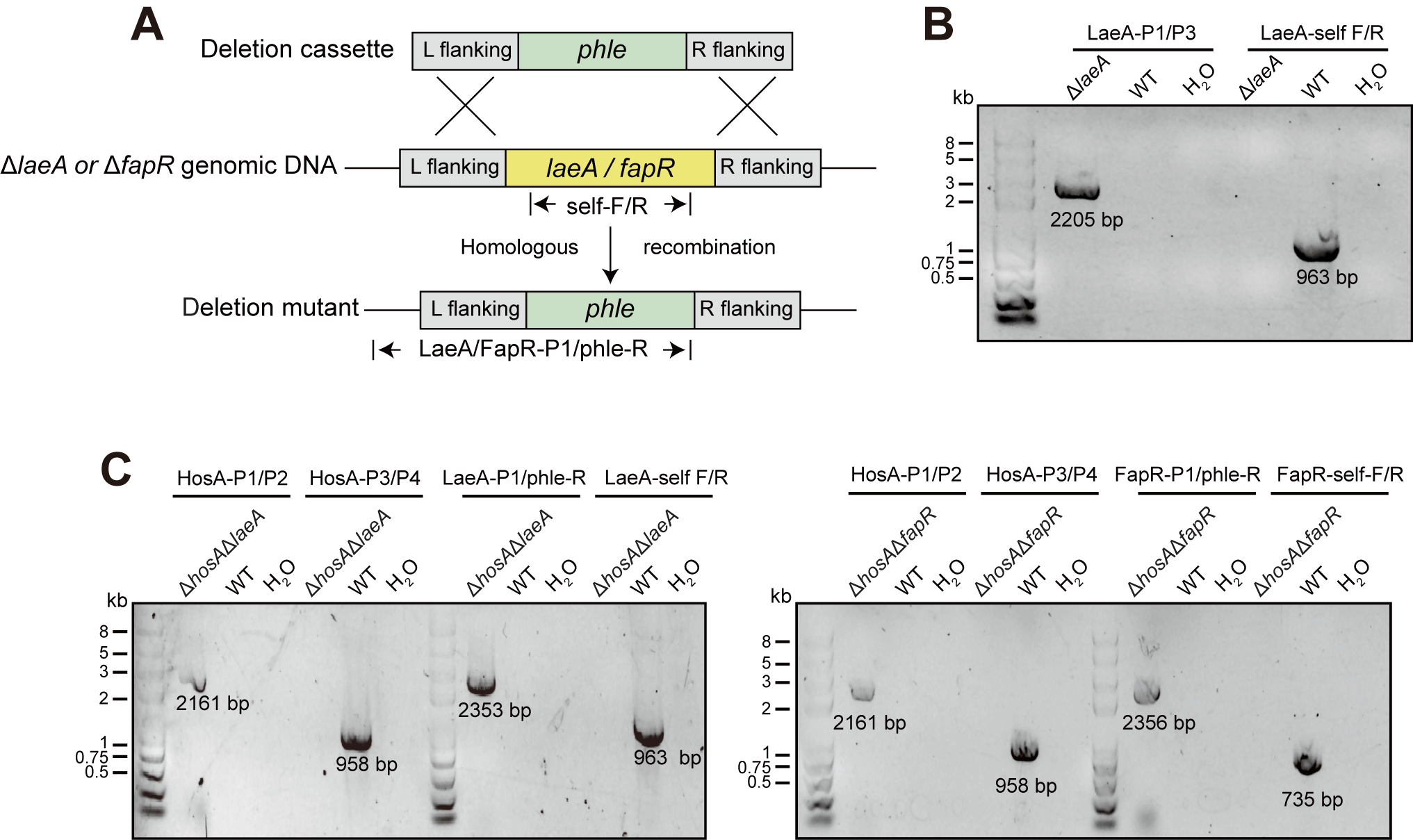

Supplement: FigS9.tif [file KVIR_A_2655064_SM2067.tif]

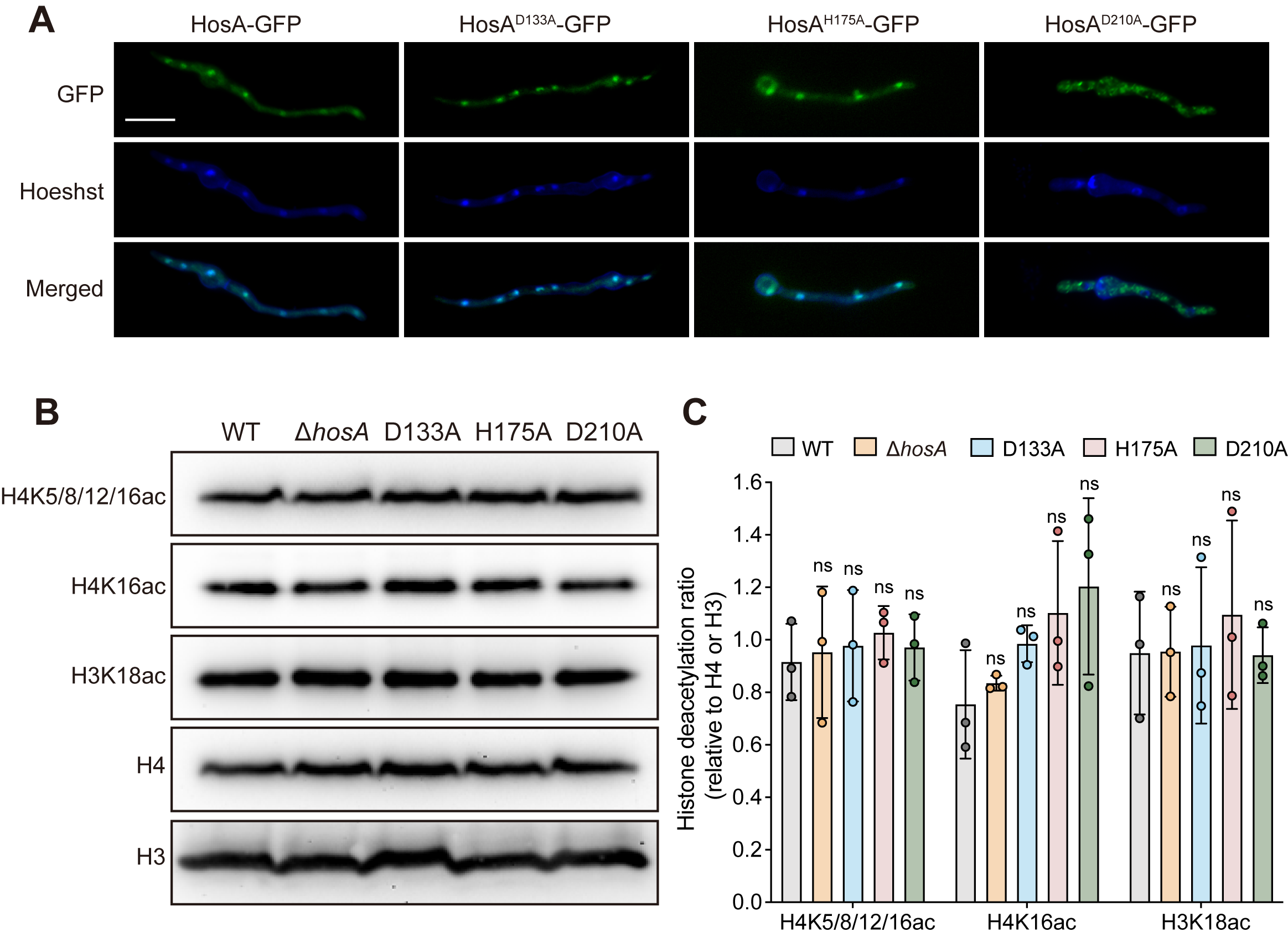

Supplement: FigS8.tif [file KVIR_A_2655064_SM2066.tif]

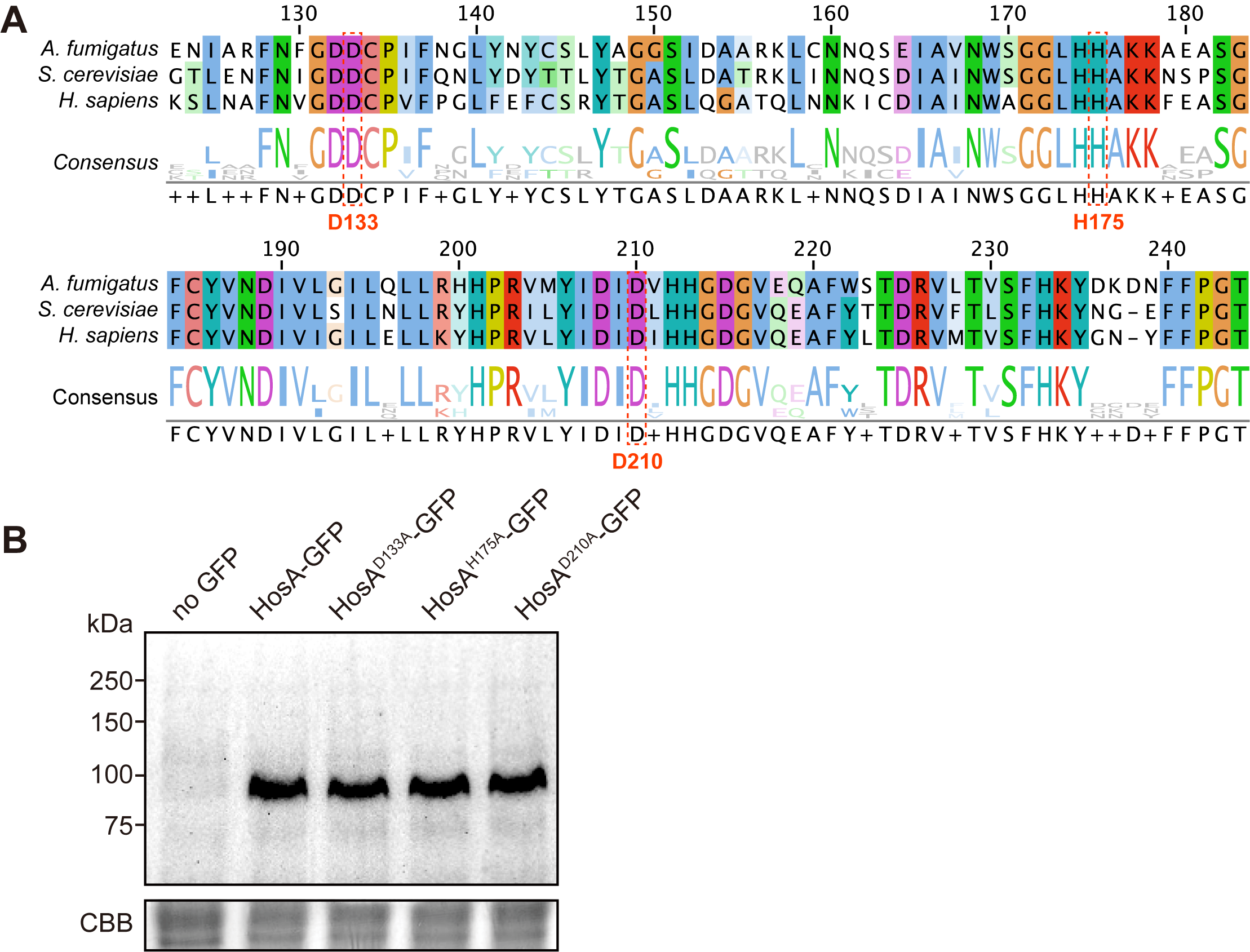

Supplement: FigS5.tif [file KVIR_A_2655064_SM2065.tif]

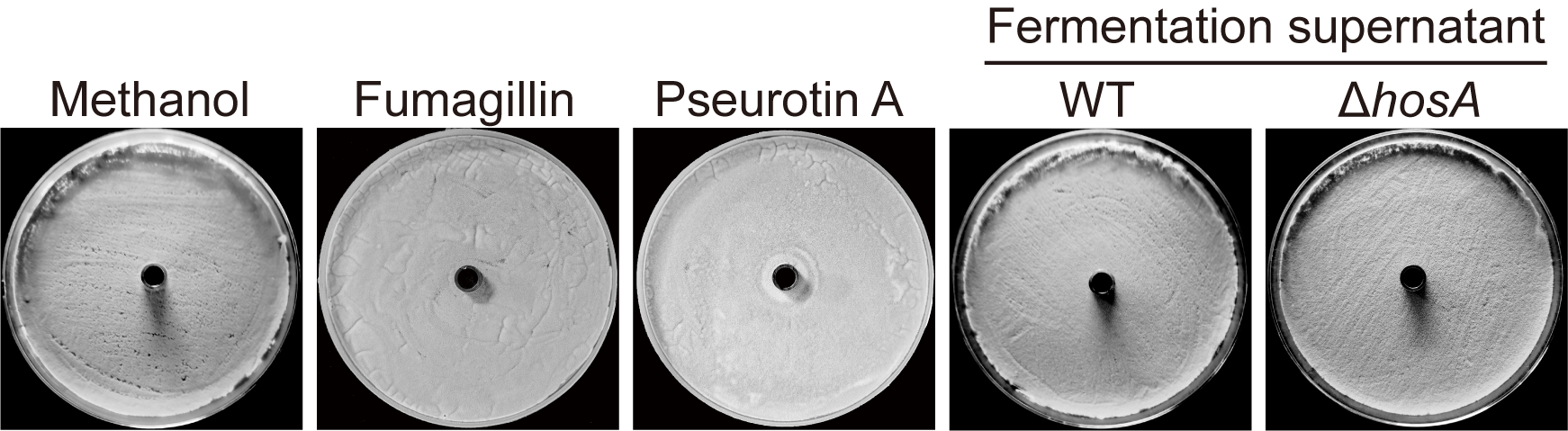

Supplement: FigS10.tif [file KVIR_A_2655064_SM2064.tif]

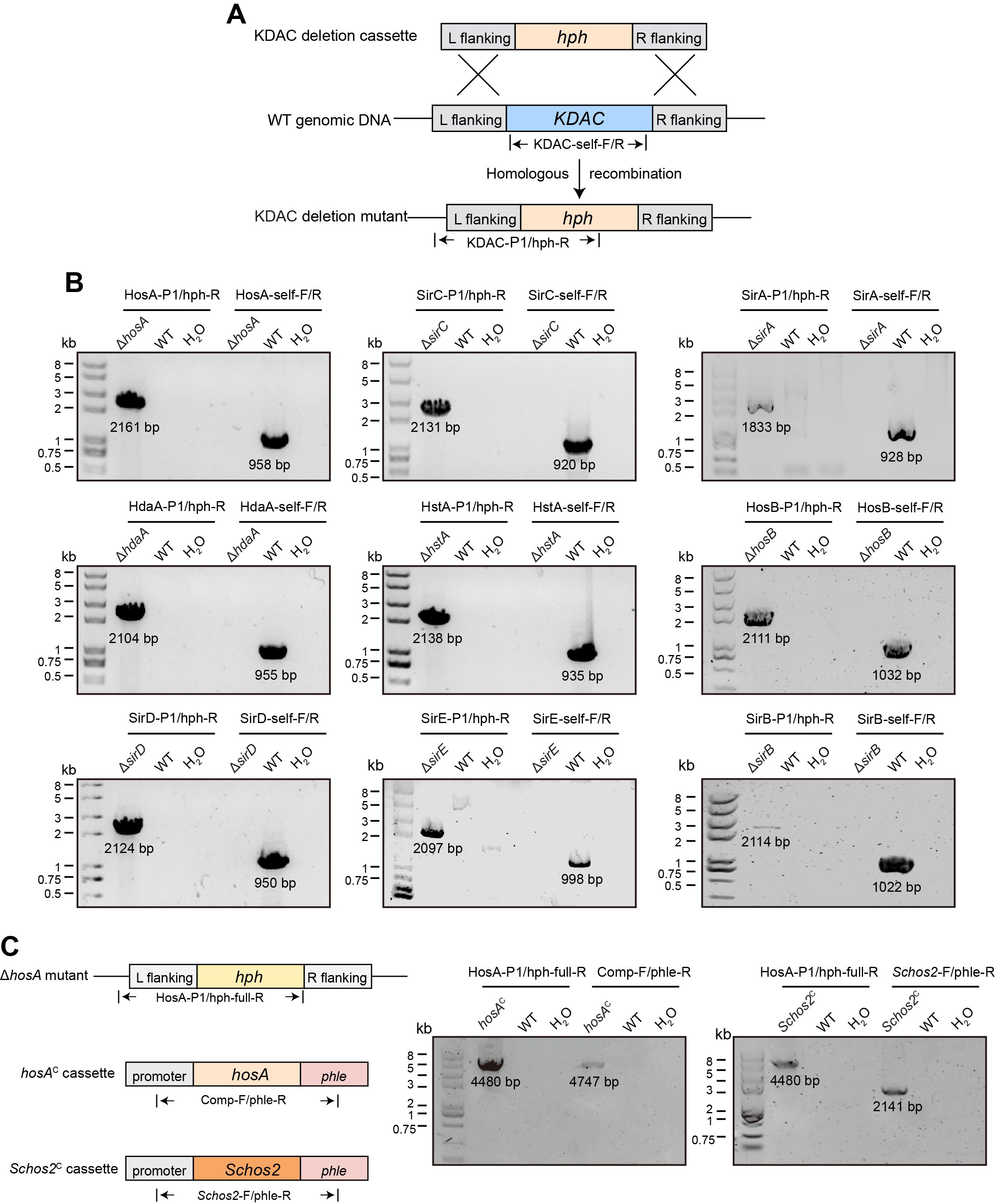

Supplement: FigS1.jpg [file KVIR_A_2655064_SM2062.jpg]

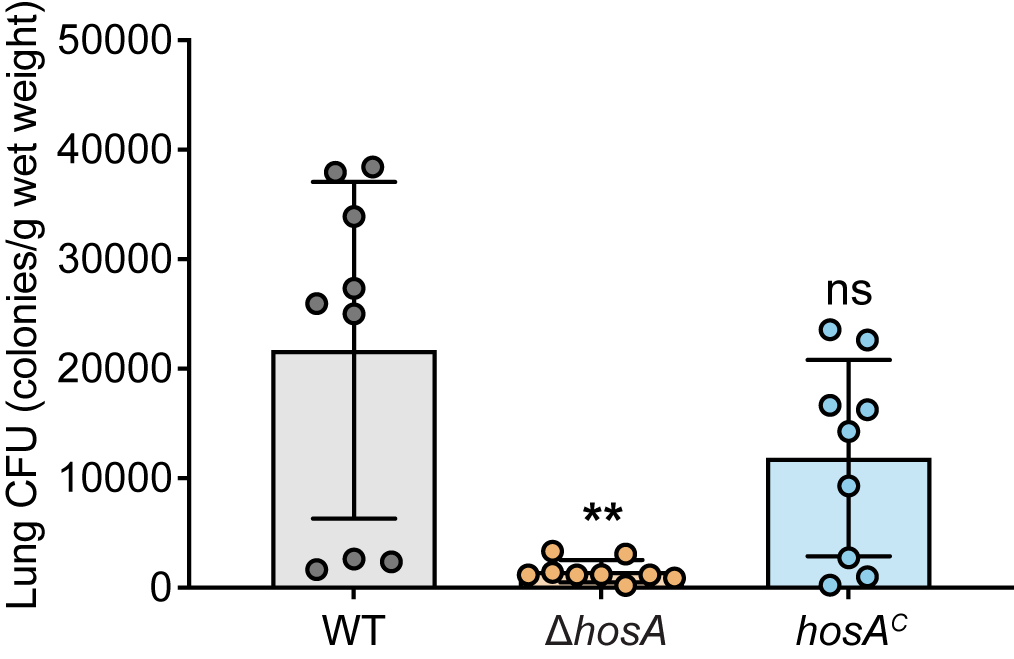

Supplement: FigS7.tif [file KVIR_A_2655064_SM2061.tif]

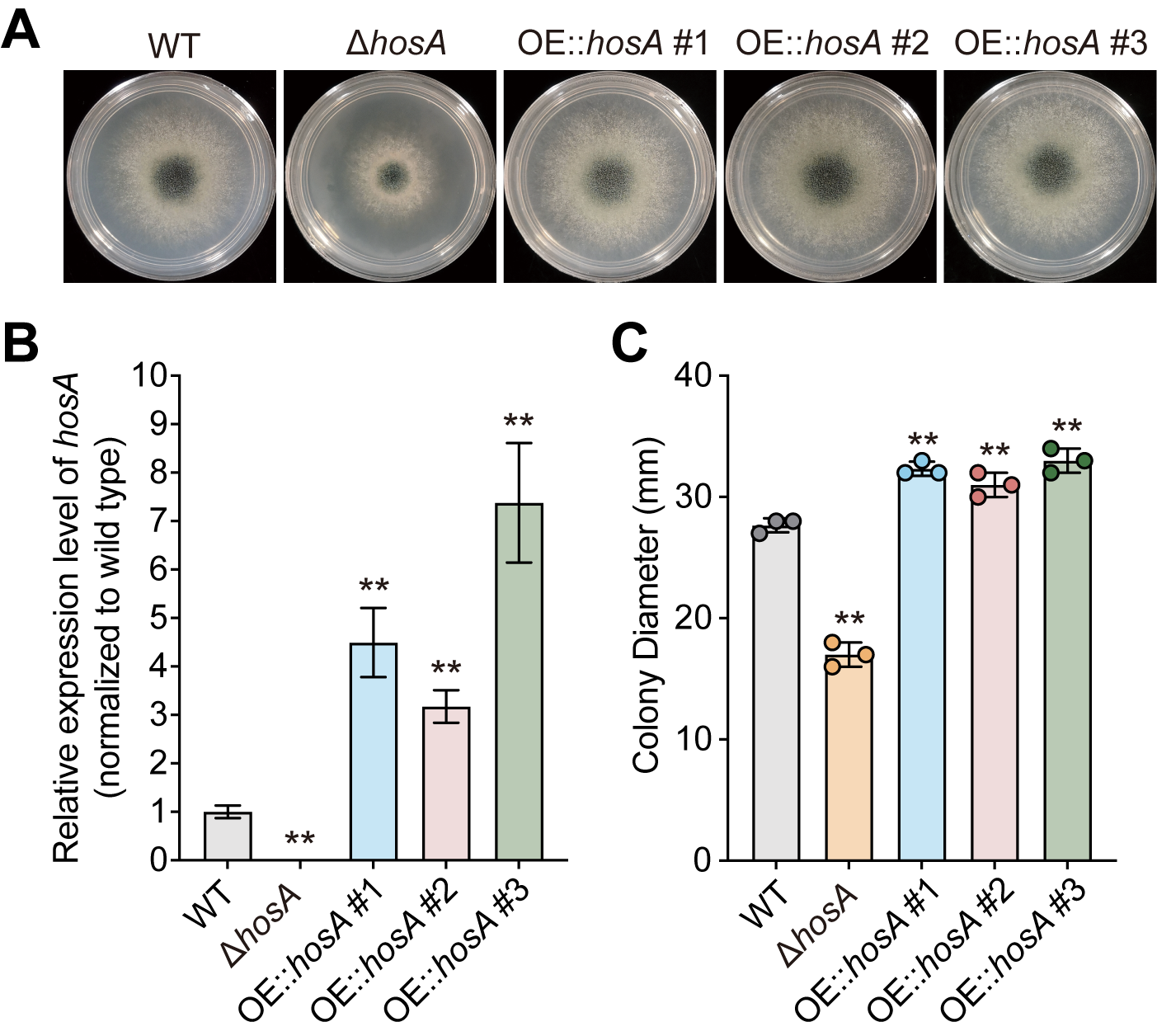

Supplement: FigS4.tif [file KVIR_A_2655064_SM2060.tif]
