# Supplementary material for: HosA-mediated epigenetic regulation of growth, virulence, and secondary metabolism in Aspergillus fumigatus
Source: Virulence. 2026 Apr 2;17(1):2655064. doi: 10.1080/21505594.2026.2655064 (PMC13078215; doi:10.1080/21505594.2026.2655064)
Supplement: 20260317_Supplementary TableS2.docx [file KVIR_A_2655064_SM2063.docx]

**Table S2. Primers used in this study.**

| **Primer** | **Sequence 5'-3'** | |
| --- | --- | --- |
| AFUB_086070-F | ggttccttgatactggagacg | |
| AFUB_086070-R | ggatgtgacgagatggcttc | |
| AFUB_086140-F | tgactgaacagcatgccg | |
| AFUB_086140-R | aaagtctcgaactcgccag | |
| AFUB_086170-F | gtggaacccgagctattacg | |
| AFUB_086170-R | caacctttgtgctcttgtcg | |
| fapI-F | tctgaagctcggtgtcaaag | |
| fapII-F | ctcgcgtttcgttggtattg | |
| fapII-R | tcgtcaacttcctttccagtc | |
| fapI-R | gtgaactggtaggacggaaac | |
| fapR-F | aattgctaggcgtgatagtcg | |
| fapR-P1 | tatcgatgttcaaccccgtg | |
| fapR-P2 | gccaagcacaccaataccaa | |
| fapR-P3 | taatcaattgcccgtctgtcactatgaagccttcgttgccg | |
| fapR-P4 | gcttacattcacgccctcctctgttgtggtctttgctcgt | |
| fapR-P5 | gcttctctggcgttgatctg | |
| fapR-P6 | aagggtgattgccaaactcg | |
| fapR-R | agcccaagtcgtgaacaag | |
| fapR-self-F | gtgagatgctcgttcgacttag | |
| fapR-self-R | ttgactcgtatggacactgc | |
| Flag-F | ctcgagggatccccgggaatg | |
| fmaA-F | ccagctatgacgaaaaggacg | |
| fmaA-R | caaacgagggaagatgggtag | |
| fmaB-F | attagccacttcttcgacctaag | |
| fmaB-R | atagccacagattgacaccag | |
| fmaC-F | tgatgggttgatattgcgagg | |
| fmaC-R | ccgttactccctgttgctg | |
| fmaD-F | gctgtccaccttaccacg | |
| fmaD-R | gtctcatccgaccagtcatg | |
| fmaE-F | cacgtcggcatctgacag | |
| fmaE-R | aacagttacacatgggacgg | |
| fmaF-F | gcgatctgacgtatatggctc | |
| fmaF-R | gtcttcggagtaattcggtgg | |
| fmaG-F | atccattcggttcatcctacag | |
| fmaG-R | gatcaactcgaccaacggg | |
| fmaKR-F | gccattccaagcaaactacg | |
| fmaKR-R | gaatgaacccgagccctatg | |
| GFP+pyrG-F | ggagctggtgcaggcgctgg | |
| GFP+pyrG-R | ctgtctgagaggaggcactgatg | |
| HosA-D133A-F | gcctgtcccatcttcaacgggttgtacaacta | |
| HosA-D133A-R | ttgaagatgggacaggcgtcgccgaaattgaaccg | |
| HosA-H175A-F | cctgcacgccgccaaaaaggccgaagccagcgg | |
| HosA-H175A-R | tttttggcggcgtgcaggccgcccgaccagttc | |
| HosA-D210A-F | atatcgacatcgccgtgcaccacggcgacggc | |
| HosA-D210A-R | cacggcgatgtcgatatacatgacgcgtgggt | |
| HosA-FLAG-P3 | cattcccggggatccctcgagagaatagagctctccccggt | |
| HosA-FLAG-P4 | ataagtagccagttcccgaaagctgattgtcttgcgtttcatt | |
| HosA-GFP-P1 | gcatttgtgtagcgaatccca | |
| HosA-GFP-P2 | acgccatggacctctatctg | |
| HosA-GFP-P3 | ccagcgcctgcaccagctccagaatagagctctccccggt | |
| HosA-GFP-P4 | catcagtgcctcctctcagacagtgattgtcttgcgtttcatt | |
| HosA-GFP-P5 | ctccttgcccgcaaccac | |
| HosA-GFP-P6 | acaccacatccgaattgacg | |
| HosA-P1 | agtttccactgctacaccca | |
| HosA-P2 | taccagtatcttgcctgcga | |
| HosA-P3 | cggcggattttaggctcaagcggttgggaaggattaggaga | |
| HosA-P4 | gttgcctagtgaatgctccggtttgctcgtttggatgggt | |
| HosA-P5 | caaaacatacaccgcccagt | |
| HosA-P6 | acaccacatccgaattgacg | |
| HosA-phle-F | actcacgtacgtctccttgt | |
| HosA-phle-R | taatcaattgcccgtctgtcacgtgacgctaccctcaacta | |
| HosA-Self-F | acgccatggacctctatctg | |
| HosA-Self-R | ttcttgttctcgagcttgcg | |
| HosA-seq-R | gacgacgaggagaggcag | |
| hph-full-F | cttgagcctaaaatccgccg | |
| hph-full-R | cggagcattcactaggcaac | |
| hph-F | cgttcatttgtccaagcagc | |
| hph-R | ctgccggtgattcgatgaag | |
| LaeA-hph-P3 | cggcggattttaggctcaagaatcgggtattggggaagaaaa | |
| LaeA-hph-P4 | gttgcctagtgaatgctccgcccttgcatgcttcaaacct | |
| LaeA-phle-P1 | ggtactgatgggccctagtc | |
| LaeA-phle-P2 | tgcctctgattacaactgcg | |
| LaeA-phle-P3 | taatcaattgcccgtctgtcaaatcgggtattggggaagaaaa | |
| LaeA-phle-P4 | gcttacattcacgccctcctcccttgcatgcttcaaacct | |
| LaeA-phle-P5 | acgggaggatgaattcgaca | |
| LaeA-phle-P6 | gcgtcacaatattctcggca | |
| LaeA-self-F | cttcccgccacttaacgttc | |
| LaeA-self-R | tctcagtaaatccggcctcc | |
| Linear-HosA-F | ttgtcttgcgtttcatttttctct | |
| Linear-HosA-R | gaggtcggaccggttggg | |
| phle-F | acgacgtgaccctgttcatc | |
| phle-R | ccatgacttccatcgtatgcc | |
| phle-full-F | tgacagacgggcaattgatta |  |
| phle-full-R | aggagggcgtgaatgtaagc |  |
| psoA-F | gtcaatttgtcgctggatgg | |
| psoA-R | tgctctttggtcacgtcag | |
| psoC-F | gaagattctggactctggcac | |
| psoC-R | aggaatgggcgaaataggtg | |
| psoF-F | tctactttggcggaatggtc | |
| psoF-R | gttttgacaccccattgctag | |
| psoG-F | gcctccatcttgattcttgaaatg | |
| psoG-R | gttgctgggtatatgtttgcc | |
| PYR4-F | tggcgttacccaacttaatcg | |
| PYR4-R | gctttcgggaactggctacttat | |
| RT-LaeA-F | gccaatggttccccaagtat | |
| RT-LaeA-R | tctagacgatcctgctcctg | |
| ScHos2-F | ttcccaaccggtccgacctcatgtctggaacatttagttatgatgtga | |
| ScHos2-R | aaaaatgaaacgcaagacaactatgaaaaggcaatcaatccactg | |
| HosB-P1 | cgcctaaatcagccactcac | |
| HosB-P3 | cggcggattttaggctcaagcgacagaagtggcatttgaac | |
| HosB-P4 | gttgcctagtgaatgctccgctttcctactgttcctgcgc | |
| HosB-P6 | gagcctacagagtttccgga | |
| HosB-P2 | gcgctctccttgtcacaaat | |
| HosB-P5 | cattctcgatccgcaacctc | |
| HosB-self-F | ttgaccgcttgcatgtatcg | |
| HosB-self-R | cgtgttcttcaggggtaggt | |
| HdaA-P1 | atcatctccgctccttctgc | |
| HdaA-P3 | cggcggattttaggctcaagacttggtcgccttaacatgc | |
| HdaA-P4 | gttgcctagtgaatgctccggcgcagaactcacttgtctt | |
| HdaA-P6 | gcgaagatgaagatggagcg | |
| HdaA-P2 | gagggcgagctcaaaagtac | |
| HdaA-P5 | gcatgcatgtctcaactttcc | |
| HdaA-self-F | ctgtgaggcgtgtcatgatg | |
| HdaA-self-R | agataacaccgttgacccgt | |
| HstA-P1 | cccatctatgccgtctccat | |
| HstA-P3 | cggcggattttaggctcaaggacgcattgttgactgagga | |
| HstA-P4 | gttgcctagtgaatgctccgggatagagccgagaactgca | |
| HstA-P6 | tcttcgggtataacgccacg | |
| HstA-P2 | tgaagagtggaaatgcgtcg | |
| HstA-P5 | cggccggtatacaattctgc | |
| HstA-self-F | gctgttctcttgactggtgc | |
| HstA-self-R | agaatcgcctcagacggaaa | |
| ScHos2-F | ttcccaaccggtccgacctcatgtctggaacatttagttatgatgtga | |
| ScHos2-R | aaaaatgaaacgcaagacaactatgaaaaggcaatcaatccactg | |
| SirC-P1 | tgcttatcgaatgccctcaga | |
| SirC-P3 | cggcggattttaggctcaagttcacgtcactgctctcgaa | |
| SirC-P4 | gttgcctagtgaatgctccgtagcgaagagtgtgcagagg | |
| SirC-P6 | tcactccttccctttggtga | |
| SirC-P2 | ccgtccgtctctgagttact | |
| SirC-P5 | cgcgggtaagatcaacgaag | |
| SirC-self-F | gcactcttaggggctggtat | |
| SirC-self-R | ctataacacccgcgtctcct | |
| SirE-P1 | caacattgatgccgtcttcca | |
| SirE-P3 | cggcggattttaggctcaaggtagcgagaggagaacggg | |
| SirE-P4 | gttgcctagtgaatgctccgccagttcaagtttggcctttc | |
| SirE-P6 | gagtatggtggtgcattgcg | |
| SirE-P2 | aaaacccttcagaacgtcgg | |
| SirE-P5 | ttccaaccaatccccgatct | |
| SirE-self-F | gcgcaggttgatttactcgt | |
| SirE-self-R | cttcggaggagttgcattcg | |
| SirB-P1 | gccttgcacagtgttagctt | |
| SirB-P3 | cggcggattttaggctcaagctggtgtgtgcaagtttcgt | |
| SirB-P4 | gttgcctagtgaatgctccgtaatgatggcgctgatgtgg | |
| SirB-P6 | acatccaatcttcatctcggga | |
| SirB-P2 | tcgatgagctgggcgatatg | |
| SirB-P5 | gcagatatcgaagcctccct | |
| SirB-self-F | tactggaggcgcggaatatt | |
| SirB-self-R | atgccgatccaattgctgtc | |
| SirD-P1 | gccattgtgctacagtctctc | |
| SirD-P3 | cggcggattttaggctcaaggcctattccaacccctctga | |
| SirD-P4 | gttgcctagtgaatgctccgttcttgttgacacgcactgg | |
| SirD-P6 | agtgacgtcgggatcaacaa | |
| SirD-P2 | acgctctactgggacttgtc | |
| SirD-P5 | gcgcagtggatccgatact | |
| SirD-self-F | tcgccaacctgatcaaaagc | |
| SirD-self-R | taggacctcggcaaacttgt | |
| SirA-P1 | cagtacgttcaaggcacacc | |
| SirA-P3 | cggcggattttaggctcaagggtgacaaaagaagccaggg | |
| SirA-P4 | gttgcctagtgaatgctccgacccattccctacagtgtgg | |
| SirA-P6 | aggcgaagttgaggatgtca | |
| SirA-P2 | gctgagttggcggaaaagaa | |
| SirA-P5 | agaagcttcctgttccgaca | |
| SirA-Self-F | gtgccactcccttcagtagt | |
| SirA-Self-R | ctgtcctcaccgtccttctt | |
