# Supplementary material for: HosA-mediated epigenetic regulation of growth, virulence, and secondary metabolism in Aspergillus fumigatus
Source: Virulence. 2026 Apr 2;17(1):2655064. doi: 10.1080/21505594.2026.2655064 (PMC13078215; doi:10.1080/21505594.2026.2655064)
Supplement: 20260317_Supplementary TableS1.docx [file KVIR_A_2655064_SM2059.docx]

**Table S1. Strains used in this study.**

| **Strain** | **Genotype** | **Source** |
| --- | --- | --- |
| A1160 | Δ*ku80*, *pyrG-* | FGSC |
| Δ*aku^KU80^* | Δ*ku80, pyrG+* | FGSC |
| Δ*hosA* | Δ*ku80*, *pyrG+,* Δ*hosA*::*hph* | This study |
| Δ*hosB* | Δ*ku80*, *pyrG+,* Δ*hosB*::*hph* | This study |
| Δ*sirA* | Δ*ku80*, *pyrG+,* Δ*sirA*::*hph* | This study |
| Δ*sirB* | Δ*ku80*, *pyrG+,* Δ*sirB*::*hph* | This study |
| Δ*sirC* | Δ*ku80*, *pyrG+,* Δ*sirC*::*hph* | This study |
| Δ*sirD* | Δ*ku80*, *pyrG+,* Δ*sirD*::*hph* | This study |
| Δ*sirE* | Δ*ku80*, *pyrG+,* Δ*sirE*::*hph* | This study |
| Δ*hstA* | Δ*ku80*, *pyrG+,* Δ*hstA*::*hph* | This study |
| Δ*hdaA* | Δ*ku80*, *pyrG+,* Δ*hdaA*::*hph* | This study |
| Δ*laeA* | Δ*ku80*, *pyrG+,* Δ*laeA*::*hph* | This study |
| *hosA*^C^ | Δ*ku80*, *pyrG+,* Δ*hosA*::*hph*, *hosA*::*phle* | This study |
| *hosA*^Schos2^ | Δ*ku80*, *pyrG+,* Δ*hosA*::*hph*, *Schos2*::*phle* | This study |
| HosA-GFP | Δ*ku80*, *pyrG-*, *hosA*::*gfp*::*pyrG* | This study |
| HosA-FLAG | Δ*ku80*, *pyrG+, hosA*::*flag*::*hph* | This study |
| Δ*hosA*Δ*laeA* | Δ*ku80*, *pyrG+,* Δ*hosA*::*hph*, Δ*laeA*::*phle* | This study |
| Δ*hosA*Δ*fapR* | Δ*ku80*, *pyrG+,* Δ*hosA*::*hph*, Δ*fapR*::*phle* | This study |
| HosA^D133A^ | Δ*ku80*, *pyrG-*, *hosA*^D133A^::*gfp*::*pyrG* | This study |
| HosA^H175A^ | Δ*ku80*, *pyrG-*, *hosA*^H175A^::*gfp*::*pyrG* | This study |
| HosA^D210A^ | Δ*ku80*, *pyrG-*, *hosA*^D210A^::*gfp*::*pyrG* | This study |
| OE::*hosA* | Δ*ku80*, *pyrG+,* Δ*hosA*::*hph*, OE::*hosA*::*phle* | This study |
